# Unlocking data sets by calibrating populations of models to data density: A study in atrial electrophysiology

**DOI:** 10.1126/sciadv.1701676

**Published:** 2018-01-10

**Authors:** Brodie A. J. Lawson, Christopher C. Drovandi, Nicole Cusimano, Pamela Burrage, Blanca Rodriguez, Kevin Burrage

**Affiliations:** 1 Australian Research Council Centre of Excellence for Mathematical and Statistical Frontiers, School of Mathematical Sciences, Queensland University of Technology, Brisbane, Queensland, Australia.; 2 Basque Center for Applied Mathematics, Bilbao, Spain.; 3 Department of Computer Science, University of Oxford, Oxford, UK.

## Abstract

The understanding of complex physical or biological systems nearly always requires a characterization of the variability that underpins these processes. In addition, the data used to calibrate these models may also often exhibit considerable variability. A recent approach to deal with these issues has been to calibrate populations of models (POMs), multiple copies of a single mathematical model but with different parameter values, in response to experimental data. To date, this calibration has been largely limited to selecting models that produce outputs that fall within the ranges of the data set, ignoring any trends that might be present in the data. We present here a novel and general methodology for calibrating POMs to the distributions of a set of measured values in a data set. We demonstrate our technique using a data set from a cardiac electrophysiology study based on the differences in atrial action potential readings between patients exhibiting sinus rhythm (SR) or chronic atrial fibrillation (cAF) and the Courtemanche-Ramirez-Nattel model for human atrial action potentials. Not only does our approach accurately capture the variability inherent in the experimental population, but we also demonstrate how the POMs that it produces may be used to extract additional information from the data used for calibration, including improved identification of the differences underlying stratified data. We also show how our approach allows different hypotheses regarding the variability in complex systems to be quantitatively compared.

## Teaser

We describe a statistically informed calibration of in silico populations to explore variability in complex systems.

## INTRODUCTION

Mathematical modeling is vital for the understanding of complex phenomena, but the use of mathematical models requires careful specification of their parameter values against available data. In many applications, model predictions can vary sharply in response to even small changes in the values of their parameters, and yet, experimental efforts to determine these values are invariably associated with either some kind of uncertainty or inherent variability underlying the processes that are being measured. In biological and physiological contexts, for example, not only are these uncertainties typically very large, but also the values of representative parameters exhibit considerable variation between different members of a population due to differences in physiology and genetics. Properly accounting for this variability using mathematical models is critical to furthering understanding in these fields (*1*).

With regard to uncertainty quantification, techniques such as Monte Carlo sampling (*2*), polynomial chaos expansions (*3*), and Bayesian approaches, including Gaussian processes (*4*), allow the impacts of uncertainty in parameter values upon model outputs (predictions) to be quantified, or parameter values and their uncertainties to be determined in response to data collected for model outputs. However, each works from the perspective of a single, immutable model with some fixed uncertainties in its inputs and corresponding uncertainties in its outputs. This approach becomes an issue when one wishes, for example, to determine which features (parameter values) in a population predict different classes of outputs or to consider the impacts of changes to the underlying model itself.

On the other hand, a very natural approach for modeling and understanding the variability within populations is the recent technique known as populations of models (POMs) (*5*–*7*). In this approach, a collection of varying individuals is represented in kind by a collection of individual models, with the idea that the collection of models exhibits the same variability as the population being modeled. Although each individual model typically differs only in terms of the values of its parameters, each remains a model in its own right, allowing subpopulations within the POM to be identified and analyzed, and the underlying model to be easily adjusted once a POM has been constructed. Somewhat related are genetic algorithms that use multiple copies of a model with differing parameter values as their organisms (*8*), although there the focus is on breeding a single model that best fits data for a single individual, and not on characterizing variability in a population.

Here, our motivating application is that of cardiac electrophysiology, although the POM technique has also been used in biomechanics (*9*) and cholesterol pharmacology (*10*, *11*) and is highly relevant to systems biology in general (*12*). The action of the heart depends on the excitable, highly nonlinear (*13*) nature of cardiac cells, which undergo a carefully controlled process of ion uptake and release in response to electrical stimulus. In addition to the temporary intake of Ca^2+^ ions that produces the cellular contraction associated with the heartbeat, control of the potential difference across the cell’s membrane also prevents it from being restimulated too quickly. The time course of the membrane potential in response to stimulus is known as the action potential (AP), and it is the AP or the important features of it (biomarkers) that are commonly recorded in single-cell experiments.

POM research has been very active in this setting, inspired by developments in neuroscience (*5*, *6*) and beginning with the sensitivity analysis studies of Sobie (*14*). An important advancement was then to calibrate these POMs in response to experimental or clinical data so that the models in these in silico populations generated outputs that were physiologically reasonable (*7*). These calibrated POMs have been used to great effect, including suggesting modifications to existing models of rabbit ventricular (*15*) and human atrial cells (*16*) required to reproduce specific data; determining the electrophysiological properties that lead to the dangerous phenomena of alternans (*17*, *18*), repolarization abnormalities (*19*), and atrial fibrillation (*20*); and characterizing the sources of the differing function of failing hearts (*21*). The technique has also been used to explore the variable response of a population to antiarrhythmic drug treatments (*7*, *22*, *23*), to the onset of ischemia in rabbits (*24*), and to the effects of hypertrophic cardiomyopathy (*25*). Most relevant to our work, calibrated POMs were used by Sánchez *et al.* (*26*) to explore the differences between patients exhibiting sinus rhythm (SR) or chronic atrial fibrillation (cAF).

To date, calibration of POMs has been achieved almost exclusively by rejecting any trialed models that produce outputs that correspond to measurable quantities falling outside the ranges of observations for those same quantities in the data set (*27*). This prevents any obviously unphysical models from being accepted into the population but does not necessarily guarantee a good correspondence with the biomarkers’ distribution in the experimental data. Selection of models according to the ranges of values observed in the data creates a feasible region that is necessarily hyperrectangular, whereas the actual multidimensional spread of experimental measurements may be a much more complex shape. In addition, it may be desirable that the selected models not only are feasible but also together exhibit key features of the distributions of experimental data, such as regions of high or low density and correlations between measured quantities. Of course, these considerations are only relevant when there are sufficient data to reasonably estimate the distributions of the measured quantities, and otherwise, range-based calibration is highly appropriate. Two recent works in cardiac electrophysiology did select parameter values by ensuring that the models together exhibited appropriate mean and SD for the experimental measures of interest (*28*, *29*), a step toward the distribution-driven calibration technique we introduce in this publication.

We extend a recent statistically informed sampling technique for POM construction (*22*), sequential Monte Carlo (SMC), to propose a new method that produces POMs directly calibrated to data distributions. As compared to Latin hypercube sampling (LHS), the uniform sampling approach typically used for range-based calibration (*27*), SMC identifies regions of the parameter space that correspond to a higher density of data and generates proportionally more samples in these locations. It also allows the complexity of the sampling problem to be introduced in a gradual fashion, relevant also when calibrating to ranges (*22*). We demonstrate not only how our calibration technique generates POMs that reproduce the distributions in experimental data but also, more importantly, how this type of calibration allows additional insights to be gained from these data. Specifically, we demonstrate how biomarker data from myocytes taken from SR and cAF atria encode almost all of the differences in ion channel expression that characterize the cAF pathology, but that distributions of the biomarkers must be used for calibration to recover them. We also show that our distribution-calibrated POMs serve as a method for parameter selection in response to data and as a tool to explore different hypotheses regarding the sources and extent of variability in unobserved properties that manifest experimentally observed variability. We finally conclude by discussing our new approach, when and how it should be used, and the implications for modeling and understanding variability in all its manifestations, both within and outside of cardiac electrophysiology.

## Experimental data and model choice

The experimental data we use to demonstrate our techniques for data-calibrated POMs were presented by Sánchez *et al.* (*26*) and consist of biomarker values measured from recorded APs for 469 cells taken from the right atrial appendages of 363 patients. These patients belonged to either one of two groups, those exhibiting standard SR and those exhibiting cAF. Thus, not only are there two data sets for which we demonstrate our calibration process, but also we are able to explore how the in silico populations calibrated to the two data sets compare. The large number of recordings in the data set makes it particularly suited to the idea of distributional calibration, because the distribution implied by the data can be meaningfully estimated.

The biomarker values used to quantify the recorded APs are the AP duration computed at 20, 50, and 90% repolarization (APD_20_, APD_50_, and APD_90_, respectively), the AP amplitude (APA), resting membrane potential (RMP), the membrane potential at 20% of APD_90_ (*V*_20_), and the maximum upstroke velocity (*dV*/*dt*_max_). More information regarding the experimental conditions under which the data were collected is available in the study of Sánchez *et al*. (*26*).

**Fig. 1:**
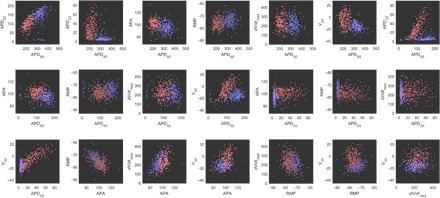
Experimental recordings of AP biomarkers show significant differences between SR and cAF. Pairwise scatterplots of each unique pair of biomarkers in the SR data set (blue) and the cAF data set (red). Clear differences in how biomarker values are distributed between the two populations are seen, especially for the APD biomarkers. Overall, the data exhibit a large amount of variability, highlighting the importance of characterizing this variability in understanding cardiac electrophysiology.

Figure 1 visualizes the spread of biomarker values for both SR and cAF APs. Clear differences in the distributions of SR and cAF biomarkers are immediately apparent, and when the biomarkers are considered individually, all of them, except for *dV*/*dt*_max_, show a statistically significant difference (*P* < 0.001) between SR and cAF myocytes (*26*). It is thus reasonable to expect that calibration of these two data sets will be indicative of the differences that underlie the cAF pathology, as compared to SR. We also note that the values of *dV*/*dt*_max_ range as high as 434 V/s, which is much larger than the typical values of 150 to 300 V/s for this biomarker in atrial myocytes (*30*, *31*).

To construct our in silico POMs, we selected the Courtemanche-Ramirez-Nattel (CRN) model (*32*) following preliminary studies that suggested that out of three potential models, it was most able to capture biomarker values seen in the Sánchez *et al*. data set. A separate benchmarking study also suggested that this model, despite being one of the first developed for human atria, predicted APDs very well for data from both SR and cAF patients (*33*). POMs were then constructed by allowing the conductances of the model’s most important currents to vary (see Materials and Methods for further details and justification). However, as will be seen later, our approach initially struggled when attempting to replicate the larger values of *dV*/*dt*_max_ in the data set and the relationship between this biomarker and APA. Modifying the search parameters to additionally include a time constant associated with the inward Na^+^ current, and allowing the conductance of this current to vary more significantly, allowed these features of the data set to be much better captured.

In addition, we later make use of data regarding ion channel remodeling under cAF from a variety of individual studies. These data are used not for calibration but solely to compare observations in the literature with predictions from our calibrated POMs.

## RESULTS

### SMC significantly improves calibration of POMs to data

We used 2000 particles to initialize our SMC sampling process (see Materials and Methods for details of the algorithm), obtaining POMs composed of 1938 unique models for the SR data and 1931 unique models for the cAF data. Using 10,000 trialed models generated using LHS (10 samples with 1000 divisions in each parameter dimension) produced 1319 accepted models for the SR data set and 1338 models for the cAF data set after calibration to biomarker ranges. We note that these numbers should not be compared as a measure of efficiency, because the SMC algorithm involves multiple model runs for each particle and solves a more difficult sampling problem that takes the distributions of the data into account.

**Fig. 2:**
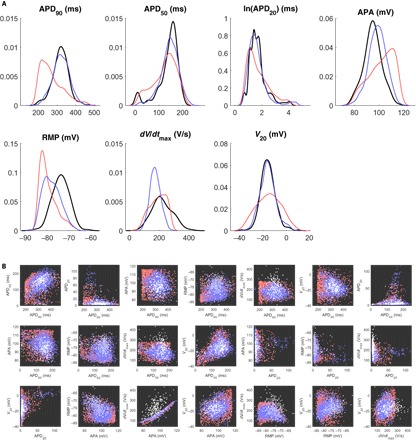
Calibration to biomarker distributions as opposed to their ranges reduces model bias. (**A**) Marginal distributions of the biomarkers in the SR data set (black) and POMs calibrated to biomarker distributions using the SMC algorithm (blue) or calibrated to biomarker ranges using LHS (red). The natural logarithm of APD_20_ values is used to better display their distribution. SMC for distributional calibration is seen to provide a significant improvement in agreement with the data. (**B**) Pairwise scatterplots of each unique pair of biomarkers in the SR data set (white) and the POMs constructed using SMC matched to distributions (blue) and LHS matched to ranges (red). The SMC-generated POM demonstrates good localization to the dense regions in the data but requires further calibration. An obvious correlation between APA and *dV*/*dt*_max_ is exhibited by the model, regardless of the sampling method used, but this correlation is not present in the data.

For the SR data set, the SMC-calibrated POMs show a significantly better degree of localization to data-dense regions in the biomarker space when compared to POMs calibrated to biomarker ranges (Fig. 2). Over the selected range of parameters, the CRN model tends to produce APD_90_ and RMP values lower than most of the data and APA values higher than most of the data, and so, these models are then overrepresented in the LHS POM, which only rejects models that fall outside of the range of the data. Our POM calibrated to data density using SMC greatly reduces the impacts of this “model bias” and also captures very well the extent of variance in the *V*_20_ values in the data set. However, there are still clear discrepancies in the distributions for RMP and *dV*/*dt*_max_, especially the latter, where neither the distribution-calibrated nor the range-calibrated POMs produce any models with *dV*/*dt*_max_ at the upper end of the range in the data. We specifically address this issue later in the paper. Figure 2B shows the constructed POMs and the data in terms of each of the different pairs of biomarkers, making it clearer how our SMC sampling approach is selecting models more concentrated in regions of higher data density. However, it is evident that a further removal of some models of the population would improve the concordance between POM and data, highlighting the importance of our refinement process that we describe subsequently. The results for the cAF data set are very similar (fig. S1).

### Refinement via selection of optimal subpopulations captures well the variability in data sets

To further improve the fit between constructed POMs and data, we include a second phase of our calibration process that selectively removes models from the SMC-constructed POMs, which we term “refinement.” Our easily approximated divergence measure, ρ, provides a means for selecting this subpopulation using a simulated annealing-type algorithm (see the “Further POM refinement” section in Materials and Methods).

**Fig. 3:**
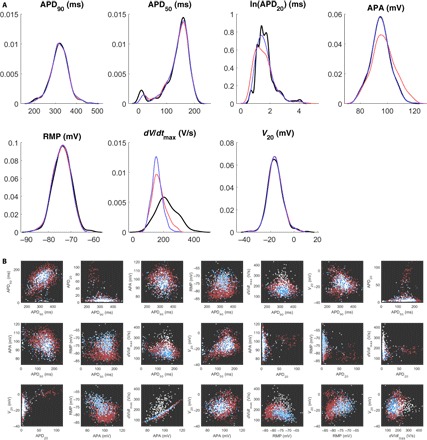
Selection of an optimal subpopulation almost fully captures biomarker variability. (**A**) Marginal distributions of the biomarkers in the SR data set (black) and POMs selected as subpopulations of the SMC-generated POM that minimized ρ (red) or $\hat{\rho }$ (blue). The simulated annealing algorithm succeeds at selecting a representative subpopulation, but the distributions of the APA and *dV*/*dt*_max_ are not quite captured. De-emphasizing *dV*/*dt*_max_ in the calibration process provides very good capture of variability in all other biomarkers. (**B**) Pairwise scatterplots of each unique pair of biomarkers in the SR data set (white) and the models from the SMC-generated POM that were accepted (light blue) or rejected (dark red) in the process of minimizing $\hat{\rho }$. Outside of *dV*/*dt*_max_, especially its relationship with APA, the features of the data are very well represented by the final POM.

For the SR data set, minimizing ρ produced a refined POM of 275 models with very good representation of variability in the data set (red line in Fig. 3), with the marginal distributions of the biomarkers matching very well apart from APD_20_, APA, and *dV*/*dt*_max_. The inability of the calibration process to capture the distributions of these biomarkers is predominantly explained by the strong correlation in the model between APA and *dV*/*dt*_max_, as seen in Fig. 2B (third row, third column). This correlation inescapably ties these two biomarkers together and prevents the refinement process from selecting models that are appropriately spread across both at once.

The inability to disentangle *dV*/*dt*_max_ from APA in the model, along with the model’s consistent underestimation of this biomarker as compared to the data, motivates the use of a second divergence measure $\hat{\rho }$ (see Materials and Methods) that reduces the emphasis on this biomarker in the calibration process. When a subpopulation that minimizes $\hat{\rho }$ is selected, the result is a POM composed of 327 models that show slight improvements in the marginal distributions of the other biomarkers and a more significant improvement in APD_20_ (blue line in Fig. 3). As might be expected, the distribution of APA values is now exceedingly well represented in the POM, because the algorithm’s efforts to minimize the divergence from the maximum upstroke velocity no longer hamper its ability to fit the distribution of the highly correlated APA. Table S1 also shows how the key statistical properties of the biomarker data are captured by our refined POM. Similarly, good performance is also achieved by refined POMs calibrated to the cAF data set (fig. S2 and table S1).

Figure 3B provides a visual demonstration of the two-phase calibration process, showing the selection of a subpopulation of SMC-selected models that corresponds well to the density of data across the biomarker space by minimizing $\hat{\rho }$. Although a large proportion of models are rejected by this process (83% for the SR data set), larger POMs can be generated as desired by using additional particles in the SMC algorithm or by modifying the “energy” minimized in the refinement process so that POMs of a larger size are encouraged. We also note that the improved performance of calibrating to our modified divergence measure $\hat{\rho }$ comes at little cost to the value of the original divergence measure ρ, as shown by table S2. For this reason, we favor POMs calibrated by minimizing $\hat{\rho }$ in the remainder of this work.

Our SMC sampling process with subsequent refinements has been seen to successfully produce POMs that accurately reflect the distributions of outputs in the data for two (SR and cAF) data sets that show significant variation between individual samples. This was achieved using a strongly nonlinear model and moderately high numbers of both variable parameters and observed biomarkers. However, the issue that our POMs show a strong correlation between the biomarkers *dV*/*dt*_max_ and APA remains, despite very little relationship between these two biomarkers in the data. We now explore whether further variability in additional parameters can explain this discrepancy.

### Variability in Na^+^ ion channel inactivation further explains the trends in upstroke velocity

The upstroke portion of the AP is controlled primarily by the inward sodium current *I*_Na_, and so, we focus our exploration of additional variability on this current. One hypothesis is that variation in the time constant of the “*h*” gate, which controls the initial inactivation of *I*_Na_, could explain the lack of correlation between upstroke velocity and APA in the data, yet implicit in the calibrated POMs. If the rate at which *I*_Na_ switches off during the upstroke is varied, then this serves to decouple these two biomarkers. For example, a fast upstroke need not necessarily also lead to a high APA if *I*_Na_ deactivates more quickly. Variability in the rate of this inactivation has been experimentally observed in human atrial myocytes (*34*).

Introducing a scaling parameter for this time constant, τ_*h*(scale)_, we performed a new calibration, with this additional parameter allowed to vary ±100% along with the channel conductances. In addition, we increased the range of *g*_Na_ so that it could take values up to +300% to give the CRN model the best chance at realizing the very high upstroke velocities seen in the data set. Refinement for this study minimized ρ, not $\hat{\rho }$, because our goal is to specifically explore whether the distribution of *dV*/*dt*_max_ in the data can be captured by our calibration process.

**Fig. 4:**
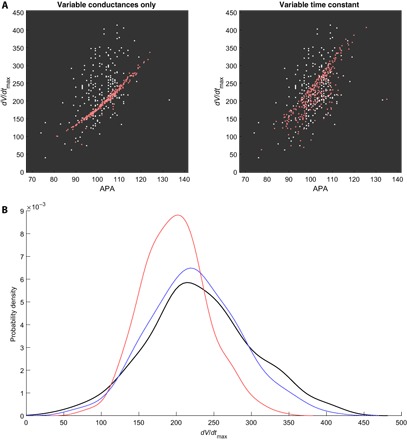
Further variance in *I*_Na_ improves the realization of *dV*/*dt*_max_ values in the cAF data set. (**A**) Pairwise scatterplot of APA and *dV*/*dt*_max_ values in the cAF data set (white) and those accepted by distribution-calibrated POMs minimizing ρ (red). Allowing variance in the time constant significantly reduces the correlation between these two biomarkers in the POM, better realizing the spread of the data. (**B**) Marginal distribution of *dV*/*dt*_max_ values in the cAF data set (black) and the calibrated POMs varying only current conductances (red) or with additional variance in *I*_Na_ conductance and inactivation time (blue). This additional variance allows our calibrated POM to almost capture the marginal distribution of *dV*/*dt*_max_ values, where the original POM fails.

The new distribution-calibrated POMs show less correlation between *dV*/*dt*_max_ and APA, although the two biomarkers still remain more strongly correlated than in the data. The result is best demonstrated for the cAF data set, shown in Fig. 4A. Furthermore, the marginal distribution of *dV*/*dt*_max_ is significantly improved (Fig. 4B), with little lost in the fit to distributions of the other biomarkers (table S2). The results for SR are very similar (fig. S3). This suggests that variance in the inactivation rate of *I*_Na_ may be an important contributor to the variability seen in AP upstrokes in the data.

### Distributional calibration produces POMs that capture important data features

Previous POM studies in cardiac electrophysiology have been almost entirely limited to varying only ion channel conductances, given their established importance to variability in cardiomyocyte behavior (*27*). This reflects the large variation of ion channel numbers across individual myocytes. Furthermore, there are less clear mechanisms for incorporating the variability associated with additional parameters controlling the time and voltage dependencies in ion channel activation/inactivation. For these reasons, we return to the original POMs where the only variable parameters are the channel conductances, and refinement is performed by minimizing $\hat{\rho }$, reducing the emphasis on *dV*/*dt*_max_. We now investigate how these distribution-calibrated POMs can be used to inform cardiac electrophysiology and hence demonstrate the advantages offered by our calibration technique.

**Fig. 5:**
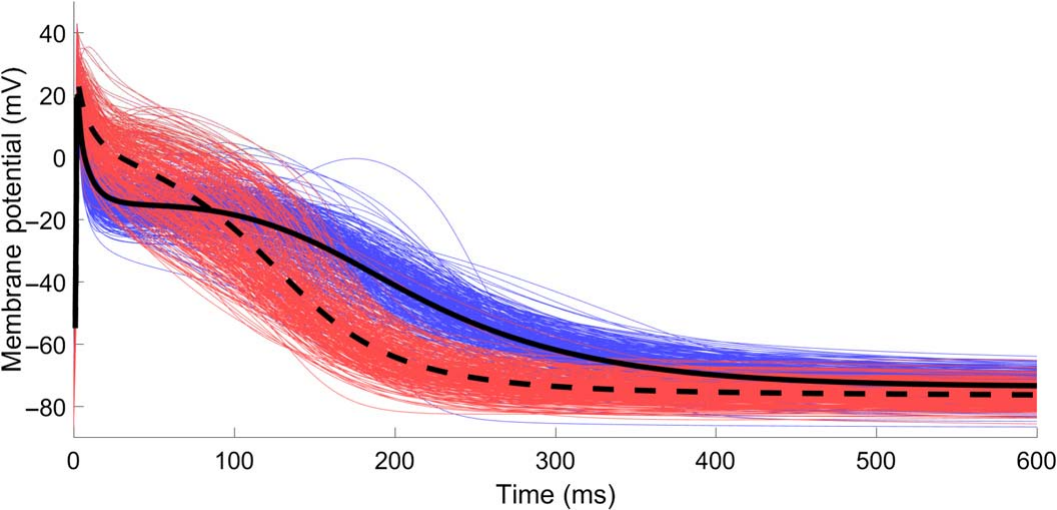
Distributional calibration captures the morphological differences between SR and cAF atrial APs. Atrial APs produced by simulation of the populations of CRN models calibrated to biomarker data for patients exhibiting SR (blue) and cAF (red). In addition, the average of all traces for the SR (solid) and atrial fibrillation (dashed) populations is displayed. The increase in AP triangulation and reduced refractory period associated with cAF is demonstrated, especially by the averaged traces.

We begin by directly considering the most important output of the populations of CRN models that we have produced, the APs, shown in Fig. 5. Significant differences between the SR and cAF populations are immediately observed. SR APs demonstrate an initial period of very rapid repolarization after the AP peak, and then an extended plateau phase before gradual repolarization back to the resting potential. In contrast, cAF APs show much less significant initial repolarization, but their lack of any significant plateau phase and overall faster repolarization produces significantly lower APDs (and hence a decreased refractory period).

These features are well known to be associated with cAF, which is characterized by far more triangular APs that lack a noticeable plateau phase and return to resting potential more rapidly than APs in healthy SR (*35*). The morphological differences between SR and cAF APs are seen in the data, with the rapid repolarization followed by plateau in the SR population implied by very small APD_20_ values and larger APD_50_ values, whereas the AP triangulation in the cAF population is seen in larger APD_20_ but smaller APD_50_ values. Calibrating to distributions naturally takes these features of the data into account, successfully selecting models that predict the appropriate morphologies. When calibrating to ranges for this data set, the models selected do successfully produce APs that show the reduced APD as associated with cAF but are less successful in predicting the accompanying differences in AP morphology [see fig. S4 and figures 2 and 3 in the study of Sánchez *et al*. (*26*)]. We note that previous studies have created additional biomarkers that can be expressed in terms of the original biomarkers, such as measures of triangulation based on combinations of different APD values, to allow more effective calibration by capturing these additional features (*21*). However, our method does not depend on identifying the important trends in data or on designing additional outputs to capture them, making it generally applicable.

Accurate prediction of the specific shapes of the APs for SR and cAF patients is important because it suggests that the differential actions of the many ionic currents that together produce the AP are being well captured by the POMs calibrated to distributions. This is critical when it comes to using these POMs for further analysis, such as considering the response of the different members of the population to drug treatments that act on specific membrane currents (*7*, *22*) or identifying the differences in underlying electrophysiology that characterize the two populations. We demonstrate these aspects in the following subsection.

### Distributional calibration produces POMs that capture key atrial electrophysiological aspects

#### Impacts of cAF-induced remodeling

We have constructed POMs calibrated to the SR and cAF data sets by varying the relative strengths of the different currents that contribute to the human atrial AP, and thus, any significant differences in parameter values selected for the two data sets suggest that changes in these currents produce the modified APs associated with the cAF pathology. Electrical remodeling of atrial myocytes that changes the densities of their different ion channels is a well-known feature of cAF and contributes to the persistence of the condition (*36*). Dobrev and Ravens (*37*) provide a review of the experimental evidence for the changes in current density associated with cAF, although further remodeling has since been experimentally identified (*31*).

Sánchez *et al.* (*26*) also compared the POMs generated for the CRN model and the models of Maleckar *et al.* (*38*) and Grandi *et al.* (*39*) when calibrated to the ranges of the SR and the cAF data by varying the six currents identified as most important to AP properties. They identified a statistically significant up-regulation of *I*_K1_ in all three models, with changes in other currents found to be model-dependent. In the case of the CRN model, Sánchez *et al.* also found statistically significant decreases in *I*_CaL_ and *I*_to_ in accordance with experimental observation (*36*). However, the observed decreases in *I*_to_ and *I*_CaL_ were quite small, and the POMs constructed failed to identify *I*_Kur_ as a significantly down-regulated current in cAF. Furthermore, *I*_NaCa_ showed a statistically significant decrease, despite Na^+^/Ca^2+^ exchanger action being known to increase in cAF-afflicted atria (*40*).

**Fig. 6:**
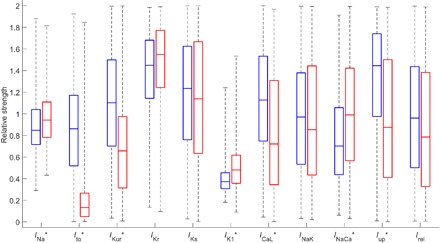
Accepted parameter values for the SR and cAF populations predict well the changes in ionic behavior associated with the cAF pathology. Box plot of θ values comprising the POMs calibrated to the SR (blue) and cAF (red) data sets. Values are expressed in relation to the base parameter values for the CRN model. Current densities that show statistically significant differences (*P* < 0.001 from the Mann-Whitney *U* test) are indicated with “*.” The currents most well known as remodeled in cAF (*I*_to_, *I*_Kur_, *I*_K1_, and *I*_CaL_) all show significant differences in the correct directions.

In contrast, our POMs calibrated to data density show many significant differences in current strengths between SR and cAF populations (Fig. 6) and do very well at identifying the currents that are known to be remodeled in response to cAF (Table 1). The distribution-calibrated POMs correctly identify the primary changes in current activity that characterize cAF (decreased *I*_to_, *I*_Kur_, and *I*_CaL_ and increased *I*_K1_), and, in the case of *I*_to_ and *I*_Kur_, also do very well in identifying the specific extent of these changes. Our POMs also correctly identify the up-regulation of *I*_NaCa_ and its extent and only miss the up-regulation of *I*_Ks_. Although up-regulation of *I*_Ks_ in cAF has been experimentally shown to be very strong (+100%) (*41*), the rather minor role that this current plays in normal repolarization makes any changes in it much less significant and thus difficult to detect through biomarker recordings. Last, our POMs suggest that the net change in atrial SERCA function (Ca^2+^ uptake) under cAF is a decrease, interesting in light of the combination of positive and negative impacts on SERCA function that have been observed experimentally (*42*, *43*). Our prediction of a net decrease agrees with the modification used by Grandi *et al.* (*39*) to represent the cAF case in their AP model.

**Table 1:** Experimentally observed changes in current density associated with cAF are well predicted by POMs calibrated to distributions. Changes in median current activities between the POMs calibrated to either the distributions or the ranges of the SR and cAF data sets, as compared with experimentally observed (Exp.) measurements of changes in current densities associated with this pathology. Experimental figures are taken from the specified references and rounded to the closest 10% to reflect the general uncertainty in their measurements and, in some cases, represent the combined result of multiple studies. The “↔” symbol indicates no significant change observed (*P* ≥ 0.01 from the Mann-Whitney *U* test for POMs). Distribution-calibrated POMs detect more of the differences in current densities that underlie the cAF pathology, correlating well with experimentally observed changes in the greatest majority of current densities.

**Parameter**	**Exp.**	**POMs (distributions)**	**POMs (ranges)**
*g*_Na_	↕* (*66*)	+11%	↔
*g*_to_	~−70% (*37*)	−85%	−51%
*g*_Kur_	~−50% (*37*)	−40%	−6%
*g*_Kr_	↔^†^ (*31*)	↔	+10%
*g*_Ks_	~+100% (*41*)	↔	↔
*g*_K1_	~+100% (*37*)	+29%	+33%
*g*_CaL_	~−70% (*37*, *67*)	−36%	↔
*I*_NaK(max)_	↔ (*68*)	↔	+10%
*I*_NaCa(max)_	~+40% (*40*)	+41%	−18%
*I*_up(max)_	↕^‡^ (*42*, *43*)	−39%	↔
*k*_rel_	↕^§^ (*42*)	↔	↔

*Peak *I*_*Na*_ current is reduced, but sustained *I*_Na_ increased.

†Decreases in mRNA levels have been observed (*37*), but no direct experimental evidence for change in cAF has yet been provided.

‡Ca^2+^ uptake is reduced by decreased Serca2a levels but increased by enhanced phosphorylation of SERCA inhibitors.

§Ca^2+^ release is increased but in a “leaky” fashion not necessarily best represented by changes to *k*_rel_ in the CRN model.

When we compared the SR and cAF POMs constructed using LHS calibrated to biomarker ranges, the results were similar to those seen in the Sánchez *et al*. study, although our POMs allowed more currents to vary in strength. Listed in Table 1 and visualized in fig. S5, the range-calibrated POMs underestimate the decreases in *I*_to_ and especially *I*_Kur_ and do not identify *I*_CaL_ as an important current in electrical remodeling under cAF. *I*_NaCa_ is also suggested to be down-regulated and small, but significant differences in *I*_Kr_ and *I*_NaK_ are identified despite these not agreeing with experimental evidence.

What we have seen is that, surprisingly, the AP biomarker data alone are enough to quantify most of the impacts on ion channel conductances caused by electrical remodeling under cAF. However, to successfully extract this information from the data set using POMs, it was required that we make use of the full amount of information contained within the data by calibrating to the distributions of these biomarker values. Our calibration technique thus serves as a means of detecting more subtle differences between multiple data sets (or stratified data), and the calibrated POMs that reflect these differences may then inform what changes in model parameters underlie them.

#### Response to antiarrhythmic treatment

Arrhythmias in the heart are typically treated by drugs that block specific ion channels, reducing the impact of the corresponding current(s) on the AP. A common target is the rapid component of the delayed K^+^ rectifier current (*I*_Kr_), which activates comparatively late in the AP and is a primary contributor to repolarization in this phase. Reducing flow due to this current hence prolongs the AP and can restore SR in patients with cAF (*44*). *I*_Kr_ was also the current chosen to explore the differential response of a variable population to drug treatment using POMs in a previous study (*7*).

**Fig. 7:**
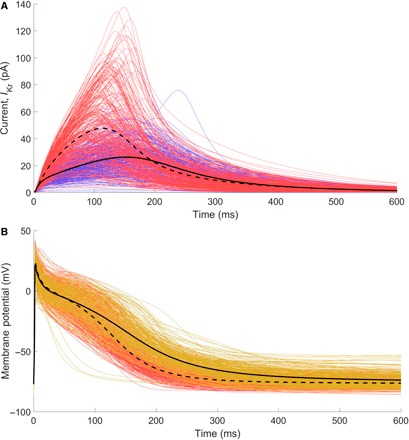
Calibration to distributions produces models that respond appropriately to antiarrhythmic treatment via *I*_Kr_ block. (**A**) Traces of the rapid component of the delayed rectifier K^+^ current for the models calibrated to the SR data set (blue, solid) and the cAF data set (red, dashed). The cAF population demonstrates an almost twofold increase in the activity of this current. (**B**) APs after treatment by 50% *I*_Kr_ block (gold) show significant AP prolongation compared to the same models without *I*_Kr_ block (red). This is also demonstrated by the averaged traces for both (black lines: treated, solid; untreated cAF, dashed). The restoration of atrial refractoriness in patients with cAF is demonstrated.

In our POMs calibrated to the SR and cAF data sets, the cAF models show significantly larger *I*_Kr_ that also activates slightly earlier (Fig. 7A), contributing to the more rapid repolarization and lack of a plateau phase in the cAF APs. However, the maximum conductance of the channel, *g*_Kr_, shows no significant difference between SR and cAF POMs (Table 1). This indicates that the increase in *I*_Kr_ activity is symptomatic of the changed AP morphology in cAF, which impacts the voltage-dependent gating behavior of these ion channels.

We explore *I*_Kr_ block via drug treatment by first pacing the models in the cAF POM until steady state (see Materials and Methods), then reducing *g*_Kr_ by 50%, and repeating the full stimulus protocol. In correspondence with the observed effects of these drug treatments, the APDs of almost all models (95%) are restored to values associated with healthy SR (Fig. 7B). This demonstrates the ability of our calibration to AP biomarker measurements to produce models that exhibit appropriate behaviors even in situations outside of those to which they were calibrated. However, there are a small number of models that predict decreased APD in response to treatment, as shown by the post–drug block APs (gold) that fall to the left of the untreated cAF APs (red), as well as a few models that repolarize to unrealistically high resting potentials (>–60 mV). One advantage of the POM framework is that these models that show unexpected behavior can be directly examined to determine the underlying causes, potentially identifying risk factors for these adverse reactions.

The models that repolarize extremely rapidly following treatment with *I*_Kr_ blocker are seen to be associated with very small values of *g*_NaK_, and thus, the unexpected behavior of these models likely stems from the selection of values for this parameter, which are too small to be physiologically realistic. This is a risk of choosing such a large (±100%) extent of variation of our parameter values, further motivating our exploration of a lower extent of variability in the following section. Nevertheless, these models do identify a potential risk factor for the use of *I*_Kr_-blocking treatments, namely, that insufficient *I*_NaK_ activity can result in dangerous further reduction of the refractory period. Reduced *I*_NaK_ under *I*_Kr_ block conditions has also been implicated as a risk factor for repolarization abnormality in a population of human ventricular models (*19*). In this case, examining the current activity in the low-*g*_NaK_ models reveals significant Na^+^ ion accumulation that occurs due to the reduced action of *I*_NaK_, which is further hampered by the reduced expulsion of K^+^ ions through *I*_Kr_. This then triggers extreme currents outward through the Na^+^/Ca^2+^ exchanger, resulting in the extra-rapid repolarization that is observed.

The models repolarizing to resting potentials that are unrealistically high are seen to be associated with incomplete deactivation of the L-type calcium channels and lower values of *g*_K1_, resulting in an imbalance of inward and outward current in the unexcited cell that gradually pushes up its membrane potential at rest. Eventually, an alternative steady state is reached where the elevated resting potential largely prevents the cell’s sodium channels from opening and the AP is severely disrupted. Parameter values that lead to this behavior will never be selected by the calibration process, because their APA and RMP values fall outside of the data. However, when outward current due to *I*_Kr_ is reduced by drug treatment and the balance of ion flow is changed, a few of the models then fail to achieve correct homeostatic balance and instead end up at this alternative steady state. The questions of whether other AP models also predict these alternative steady states and whether drug treatments can cause individual atrial cells to develop disrupted balances of ion flow at rest (compensated for by their neighbors) are beyond the scope of this paper.

The drug block case study can thus be seen as a means of further calibrating the generated POMs by testing the ability of all of the models selected to continue predicting reasonable AP curves when subject to established treatments. This is important given the tendency for currents to compensate for one another, resulting in model behaviors that only become manifest subject to this type of further interrogation. In the previous study of Britton *et al.* (*7*), albeit using a different AP model, calibrating to biomarkers recorded for multiple different pacing frequencies was seen to be sufficient for avoiding the selection of models that exhibit unphysical responses to drug block.

#### Parameter selection in response to variable data

For building or modifying AP models, experimental recordings of current actions and the APs themselves are, of course, essential for the appropriate selection of parameter values. However, the variability present in these recordings makes the selection of model parameters in response to experimental data difficult. Typically, these recordings are averaged, providing single traces for currents and APs that can then be calibrated to by inspection or with curve-fitting algorithms. However, this ignores a lot of information present in the full data set and can raise the question of which parameter values to use when AP models are known to permit multiple sets of parameter values that all lead to an equivalent AP trace (*45*).

POMs calibrated successfully to data density represent a set of models that all correspond in a sense to some portion of the data. Thus, the entirety of the data is reflected in the selection of parameter values across a distribution-calibrated POM, providing a potentially more robust means of selecting an individual set of parameter values for an AP model in response to that data. This is achieved by averaging in the space of parameter values, as opposed to in the space of measured outputs. In the case of this work, the set of parameter values selected for both the SR and cAF POMs has a rather regular distribution (no obvious bimodal behavior or obvious correlation structures; see fig. S6), and so, we consider each variable parameter individually and take the median of its value across all models in the POM, producing two modified CRN models, one each for the SR and cAF data sets.

**Fig. 8:**
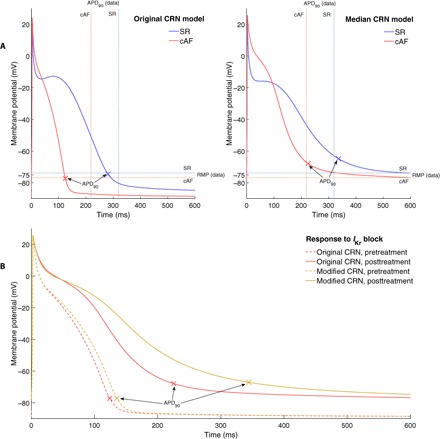
Selection of parameter values using distribution-calibrated POMs produces updated models that correspond to provided data. (**A**) AP curves for SR (blue) and cAF (red) as predicted by the original and median CRN model. Experimental calibration via POMs produces a model that fits very well the general trend of the data (mean values of biomarkers from the data indicated by dashed lines). (**B**) cAF APs before (red) and after (gold) treatment via *I*_Kr_ blocker for the original (dashed) and modified (solid) CRN model. Our median CRN model predicts the antiarrhythmic effects of *I*_Kr_ block (via APD prolongation), whereas the original CRN model predicts little response.

Figure 8A compares our “median” CRN models to the baseline CRN model and the baseline CRN model modified for cAF using the experimentally informed adjustments to its parameters listed in Table 1. The original CRN model is seen to significantly underestimate the RMP, predicting a value of −85.7 mV for SR, as compared to −73.8 mV for the mean of the SR data (−88.7 mV versus −78.4 mV for cAF). The APD is similarly underestimated, especially in the case of cAF, where the original CRN model (with cAF-adjusted parameters) predicts an APD_90_ of only 121 ms, as compared to a mean value of 216 ms in the data. In contrast, our median CRN models for SR and cAF do much better at reflecting the values for these biomarkers seen in this particular data set (RMP, −74.7 mV for SR and −78.4 mV for cAF; APD_90_, 335 ms for SR and 223 ms for cAF).

These differences are explained by the parameter values selected for our POMs, which featured many models with much lower values of *g*_K1_ and increased values of *g*_Kr_, as compared to the original published values for the CRN model. These are both late-stage repolarization currents and so can somewhat compensate for one another in terms of APD, but *I*_K1_ is a much more significant controller of RMP. Thus, its reduction is necessary to allow the CRN model (which, as stated, tended to underestimate RMP) to produce APs that corresponded with the data. As a result of the specific balance of these two currents, APD_90_ values were also increased.

The increased importance of *I*_Kr_ predicted by our median CRN model (for both SR and cAF) also makes it more predictive of the effects of drug block of this current. Figure 8B compares our median model with the original CRN model, where it can be seen that our calibrated model demonstrates a restoration of APD to SR levels, whereas the original CRN model shows very little APD prolongation at all. In cAF, the original CRN model predicts a further decrease in relevance of *I*_Kr_ for repolarization (*46*), whereas the models selected for our calibrated populations show an increased magnitude of this current in cAF (Fig. 7A). This agrees with the known efficacy of treatments targeting this current for the restoration of healthy SR in patients with cAF (*44*).

The ability of our median model to capture well the results of drug block treatment, despite no data regarding drug block used in the calibration process, leads us to suggest that our technique not only allows the selection of parameter values that better match provided data but also can result in models that are more generally predictive. This is of particular importance in fields such as cardiac electrophysiology, where very different sets of parameter values can produce very similar APs and further measures may be necessary to differentiate between these multiple sets of parameter values.

### Distributional calibration can inform the extent of variability in parameter values

Calibrating to distributions means that the variability in a supplied data set can be expected to be explicitly captured by constructed POMs, so long as the model across the specified parameter space is capable of generating outputs that match the data. This means that by using divergence measures, such as ρ and $\hat{\rho }$, the differential ability of various parameter spaces to capture variable data with POMs can be considered. The most obvious application of this technique is to explore the level of variability in parameter values needed to explain the data.

Our studies on the Sánchez *et al.* data set have used a large variation in ion channel conductance values (±100%), following previous studies (*7*, *17*, *26*). Working with such a variation in parameter values carries the risk of selecting extreme values that are not physiologically sound (for example, values close to − 100% that essentially switch off an entire current), and there is some suggestion that a level of variability, such as ±30%, is more appropriate for cardiac ion channel conductances (*26*, *27*, *47*). We therefore seek to answer the question of whether 30% variability sufficiently explains the variation in the data set we calibrated to.

**Fig. 9:**
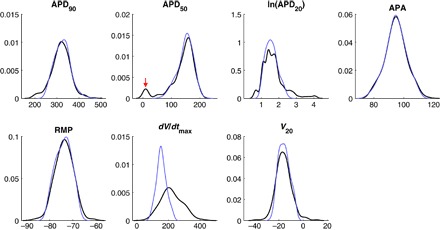
Variation of ±30% in current densities underestimates biomarker variance in the SR data set. Marginal distributions of the biomarkers in the SR data set (black) and distribution-calibrated POM using ±30% variance in ion channel conductances (blue). A reduced search space is still able to recover the general distributions of all biomarkers except for *dV*/*dt*_max_, but the full extent of variation in the APD biomarkers and *V*_20_ is not present in the calibrated POM. Notably, the very low APD_50_ values recorded for some patients are completely unrepresented in the POM (arrow).

To select the most relevant portion of the parameter space, we take parameter values ± 30% around the values selected for the median CRN models in the previous section. Applying our SMC sampling algorithm and subsequent refinement, we obtained 254 models calibrated to the SR data set and 215 models calibrated to the cAF data set, the minimum allowable number of models in both cases. Neither of these POMs succeeded in fully capturing the variability in the data set, resulting in divergence measures that compare unfavorably to those obtained using the full ±100% variation in cell properties (table S2). Examination of the marginal distributions of the biomarkers for the SR ±30% POM reveals that it does successfully capture the general distributions of the data but fails to show the same extent of variance (Fig. 9). Similar results are also seen for the cAF ±30% POM (fig. S7).

Furthermore, the secondary mode in the distribution of SR APD_50_ values is completely unrepresented by the POM constructed with reduced variability. This peak most likely corresponds to cells that repolarize more than 50% during the phase of immediate repolarization, driven primarily by the rapidly activated outward currents *I*_to_ and *I*_Kur_. This results in a cluster of very small APD_50_ values that are separate from most of the cells, which only reach 50% repolarization after the plateau phase. General underestimation of biomarker variability in the POM could potentially be explained by measurement error associated with the biomarker data, but the inability to produce models that populate this peak implies that additional variance in cell properties, at least those relevant to *I*_to_ and/or *I*_Kur_, is required to produce models that exhibit sufficient early repolarization. Coupled with the general underestimation of biomarker variance in the POM, these results certainly imply that additional variability in ion channel conductances beyond ±30% underlies the variability in biomarkers that is seen in these data sets.

## DISCUSSION

Population-based modeling is a powerful technique for allowing deterministic mathematical models to explore and characterize the variability inherent in complex systems (*1*). This includes the use of multiple regression techniques performed on synthetic populations (*14*, *48*, *49*) and, when data are explicitly available, calibrated POMs (*27*). Previously, calibration of POMs has typically been performed by ensuring that all relevant model outputs fall within the ranges of the data (*7*, *24*, *26*). Although this is a perfectly reasonable approach, especially when a low number of experimental samples are available, it does ignore other information inherent in the data and does not strictly guarantee that the models selected will produce outputs that correspond to those seen in the data. Our presented calibration technique estimates the underlying distribution of outputs represented by the data and calibrates to this distribution using a combination of SMC (*22*) and a simulated annealing-type algorithm.

We have demonstrated the efficacy of our technique on a pair of data sets from cardiac electrophysiology, a field that has seen a great deal of POM research following the pioneering studies of Prinz *et al*. (*5*) and Marder and Taylor (*6*) in neuroscience. The data used were composed of biomarker values collected for atrial myocytes from hearts exhibiting healthy SR or the cAF pathology (*26*), offering two sets of data to test the calibration technique and the opportunity to compare the models selected to represent healthy and afflicted hearts. The use of SMC to sample according to the distribution of data was found to improve the correspondence between POMs and experimental observations over LHS matched to ranges, but the approximate nature of the technique left room for further improvement. A subsequent refinement technique to select optimal subpopulations of the SMC-constructed POMs was able to produce in silico populations that reflected extremely well the variability inherent in the data distributions.

Our demonstration of our calibration technique operated from the assumption that variations in the ion channel conductances were capable of explaining the variability seen in experimental studies, consistent with previous works in the POM literature (*27*). However, under this assumption, we found that the POMs produced by our calibration technique could not match the distribution of *dV*/*dt*_max_ values as well as other biomarkers, especially the relationship between this biomarker and APA. To further address this issue, we considered the effects of incorporating further variability into the behavior of *I*_Na_. We did this by introducing an additional variable parameter, namely, the time constant of the gating variable that controls the initial inactivation of this current. This further improved the concordance between the data and our calibrated POMs in terms of these biomarkers, although this could not completely capture the nature of their relationship. This suggests that variance in *I*_Na_ inactivation rates may contribute a not insignificant portion of the variability seen in the overall excitation behavior of atrial myocytes but is insufficient to wholly explain it. Moreover, it demonstrates that the inability of our original calibrated POMs to capture the *dV*/*dt*_max_ distribution is most likely the result of our assumptions regarding the sources of variability in the problem being too restrictive and not due to any issue with the calibration process itself. This shows how our calibration technique can be seen as a tool for investigating different assumptions regarding where variability lies in a complex system or the relative importance of variability in the different components that make up these systems.

Our approach generates models that predict very well the morphologies of different types of APs associated with SR or cAF. The cAF models also demonstrate a more realistic response to antiarrhythmic treatment [AP prolongation in response to *I*_Kr_ block (*44*)], as compared to the original CRN model. These benefits remained even when the population of models was averaged to produce a single model, demonstrating the efficacy of the technique for the more general problem of selecting parameter values in response to variable data. Taking into account the variability in data in this fashion, as opposed to simply averaging it and fitting parameters to the result, is of particular importance when a system (and its associated mathematical model) is complex, such as in our example of cardiac electrophysiology, where the ability of currents to compensate for one another allows highly similar APs to be produced by very different balances of constituent currents (*7*, *50*).

Furthermore, calibration that takes more of the features of data into account may be able to resolve more subtle differences between data sets, making it a powerful approach for identifying the potential causative factors that produce these differences. We have demonstrated this usage of our technique by calibrating POMs to a pair of AP biomarker data sets. Although these biomarkers summarize the characteristic features of the AP, they do not provide much in the way of direct information regarding the activities of the different currents that constitute the AP. Nevertheless, using only these biomarker data (but only by calibrating to distributions), we found that our resultant POMs for SR and cAF were unexpectedly good at identifying the changes in ion channel activity that characterize the cAF pathology. This was validated by comparison with a suite of studies in the literature specifically quantifying the effects of electrical remodeling on individual currents in cAF (*31*, *37*). Differences in the uptake of Ca^2+^ by the sarcoplasmic reticulum were also identified, despite this being an internal current that does not directly contribute to the AP (*32*). We suspect that the contribution of the internal calcium dynamics to the current through the Na^+^/Ca^2+^ exchanger allows them to be partially identified, even when calibrating only to AP biomarkers, but this point requires further investigation beyond the scope of this paper.

With the ability of our distribution-calibrated POMs to identify the trends in model parameters that underpin specific sets of observable data, we suggest that POMs calibrated by our technique could also be used to identify differences in underlying behavior that explain, for example, pathological conditions that are less understood and not limited to cardiac electrophysiology. This same thought also extends to improved identification of any differences that underlie artificial stratifications of a data set (for example, differences in physiology that correspond to factors such as gender or age) (*51*, *52*).

When constructing POMs without experimental evidence for the extent of variability in parameters, the choice of parameter space to sample is open. Our primary study used a large space of parameter values (±100%) to give the model the best opportunity to simultaneously fit the two different sets of data and hence demonstrate our technique. However, we also considered the effects of choosing a smaller parameter space (±30%) (*21*, *26*, *47*), attaining results that imply that ±30% variance in primary current conductances is insufficient to explain the experimental data, and thus that further variation in these cell properties or others is expected. This sort of exploration into appropriate levels of variability is particularly important in fields such as cardiac electrophysiology, where the variable cell properties are difficult to directly measure and the extent of variability is not well established (*53*). This analysis requires the ability to calibrate POMs to distributions so that the comparisons between parameter spaces of different sizes are unaffected by model bias.

There are several circumstances under which our calibration process might fail or might be considered inappropriate. First, calibrating to distributions requires sufficient data in the data set to form a reasonable approximation to the underlying distribution. When the sample size is insufficient compared to the variance to suggest a specific distribution with any real confidence, obviously distributional calibration is inappropriate and calibration based on range statistics is very reasonable. However, even in these cases, a benefit can potentially be gained by enforcing that data are spread evenly across its range, although we have not investigated this in this paper. Second, successful calibration can only be achieved when the model used to construct POMs is capable of producing outputs that correspond to the range of values seen in the data. This depends strongly on the variability assumed to be present in the model, as we have seen here with the inability to capture the distribution of *dV*/*dt*_max_ values when varying only ion channel conductances. However, this does demonstrate the usefulness in considering the distribution of the data and how well-constructed POMs fit to it using measures such as ρ, in that it suggests when assumptions regarding variability (or even the models themselves) might need to be reconsidered. The varying ability of different models or patterns of variability to successfully produce POMs calibrated to a given data set also serves as a means of comparing and benchmarking them. Last, the reliance upon a transformed target density in the SMC algorithm might potentially cause it to select models that do not accurately capture the distribution of outputs in the data, although on our test problem, the method has been seen to perform very well. In the worst case, “naive” POMs composed of large numbers of models can be constructed by sampling the search space uniformly (such as through LHS) and then refined by our simulated annealing process to select the subpopulation of these that best matches the data.

In conclusion, our presented calibration technique allows data sets to be thoroughly mined to produce informative and predictive POMs that capture the variability between individual members of a population. The benefits of successfully accounting for this variability are well established (*1*). We have shown how our technique allows different assumptions regarding the sources (and extents) of variability in a system to be explored, even when some sources of this variability are indirect or have not been experimentally quantified. We have also demonstrated how calibrating specifically to data distributions allows much better differentiation between multiple sets of data, shedding light on the changes in underlying properties that explain differences in higher-level observables that can be experimentally measured. In this case, differences in AP biomarkers proved sufficient for predicting most of the changes in ion channel activity that characterize the cAF pathology but only when calibrating to the distributions of these biomarkers. Finally, we also demonstrated how the models selected by our calibration technique not only agree with the data used for calibration but also are more generally predictive, as shown in this case by a much better representation of the SR and cAF morphologies, and the effects of antiarrhythmic treatment. We recommend our technique for any systems where variability is expected to be present, a mathematical model is available, and there are sufficient data to make calibration to distributions feasible.

## MATERIALS AND METHODS

### Atrial AP model

The CRN model uses 21 coupled ordinary differential equations to simulate, among other things, the activation and inactivation of nine different sarcolemmal ion channels, as well as the actions of the sarcolemmal Ca^2+^ pump, Na^+^/K^+^ pump, and the Na^+^/Ca^2+^ exchanger, which all contribute to the flow of ions in or out of an atrial cell and thus to the changes in membrane potential that create the AP. Ion channels are modeled using a Hodgkin-Huxley–type formulation (*54*), with combinations of gating variables representing the presence/absence of activators/inhibitors that determine channel availability. Ca^2+^ uptake into the network sarcoplasmic reticulum, release from the junctional sarcoplasmic reticulum, and the transfer (active or leak) between these two compartments are also represented. For full details of the model, including the specific forms of each of its differential equations, see the study of Courtemanche *et al*. (*32*).

We simulated the CRN model using MATLAB’s ode15s routine, with a maximum step size of Δ*t* = 1 ms. Biomarkers were measured from output *V*(*t*) curves after discarding any APs that failed to excite above −30 mV, failed to repolarize to a value of RMP + 0.1 APA, or exhibited spontaneous depolarizations (judged as subsequent peaks after repolarization to the aforementioned value). Following Sánchez *et al*., the model was paced until it reached steady state (≤1% change in all state variables) and then 90 more times, using a stimulus of 2 ms of −2210 pA, approximately twice the diastolic threshold for the base model. Temperature and external ion concentrations were adjusted to match the experimental conditions (*T* = 309.65 K, [Na^+^]_o_ = 149.42 mM, [K^+^]_o_ = 4.5 mM, [Ca^2+^]_o_ = 1.8 mM). No other parameters were modified from their values in the originally published version of the model.

The process of simulating the CRN model (with input parameters θ) and the subsequent calculation of biomarkers from the resulting AP are denoted by M, and the output biomarkers are denoted by **y**, such that

y=M(θ) (1)

### Populations of CRN models

POMs were constructed here by varying a set of inputs to the CRN model, namely, the current densities of the fast Na^+^ current, the five outward K^+^ currents (transit outward, ultra-rapid delayed rectifier, rapid and slow delayed rectifiers, and the inward rectifier), the L-type inward Ca^2+^ current, the Na^+^/K^+^ pump and Na^+^/Ca^2+^ exchanger, and the maximal rates of uptake and release of Ca^2+^ inside the cell by the sarcoplasmic reticulum. Following the notation used in the original CRN paper, the set of inputs is here denoted θ = (*g*_Na_, *g*_to_, *g*_Kur_, *g*_Kr_, *g*_Ks_, *g*_K1_, *g*_CaL_, *I*_NaK(max)_, *I*_NaCa(max)_, *I*_up(max)_, *k*_rel_), where *g* is the maximal conductances of the different currents, *I*_(max)_ is the maximal actions of pumps and exchangers, and *k*_rel_ is the conductance of the ryanodine receptors that release Ca^2+^ from the sarcoplasmic reticulum. These were the same currents varied by Muszkiewicz *et al*. (*16*) in their construction of POMs for human atria. Sánchez *et al.*’s study (*26*) varied only the six currents they identified as having a significant impact on biomarker values (*55*) and thus did not include *g*_Na_, *g*_Kr_, *g*_Ks_, *I*_up(max)_, or *k*_rel_.

Following Sánchez *et al*., POMs were constructed using a search space of ±100% from the base parameter values for the CRN model. However, whereas their work selected trial points using the sampling method underlying Fourier amplitude sensitivity testing (*56*) and then rejected those that did not produce APs with biomarkers falling within experimentally observed ranges, we used the method described subsequently to produce POMs that not only corresponded to the spread of the experimental data but also reproduced its distributional features.

### Biomarker joint distribution estimation

Distributional calibration first requires estimating the distribution represented by the data, *p*(**y**). This was achieved here by multivariate kernel density estimation, which creates a smooth distribution by summing over a series of multivariate Gaussians centered at each of the *N* individual data points

$$p(y)\approx \frac{1}{\text{det}{\left(H\right)}^{-1/2}N{\left(2\pi \right)}^{-N_{b}/2}}\sum_{i=1}^{N}e^{-1/2{\left(y-{\tilde{y}}_{i}\right)}^{T}H^{-1}(y-{\tilde{y}}_{i})}$$ (2)

where *N*_b_ is the number of biomarkers (seven in this case), $\tilde{y_{i}}$ are the individual points of biomarker data, and **H** is the bandwidth matrix, a parameter of the density estimator that controls the extent and direction of smoothing. When the distribution to be estimated is normal with unit variance, the optimal bandwidth can be shown (*57*) to be

$$h_{\text{opt}}={\left(\frac{4}{N(N_{b}+2)}\right)}^{\frac{2}{N_{b}+4}}$$ (3)

which motivates a choice of bandwidth matrix

$$\begin{array}{cc} H_{ij}=h_{\text{opt}}\;\sigma_{i}^{2} & \text{if}\;i=j\\ =0 & \text{if}\;i\neq j \end{array}$$ (4)

Thus, the extent of smoothing is weighted in each dimension in terms of the variance in that biomarker observed in the data set, but correlations between biomarkers are ignored in the choice of **H.** Note that the estimated density itself still attempts to account for dependencies between biomarkers. The choice to use a diagonal bandwidth matrix tends to be sufficient in practice (*58*).

Another alternative to multivariate kernel density estimation is to approximate *p*(**y**) by combining the marginal distributions of each biomarker with a Gaussian copula to approximate their interdependencies. However, for the atrial data sets we use to demonstrate our calibration technique, this approach was found to be less effective and so is not discussed further here.

APD_20_ readings in the SR data set were predominantly clustered at low values but with a considerable range. To improve the performance of the kernel density estimation (recalling that the bandwidth was selected as optimal for normally distributed data), the APD_20_ values were first logarithmically transformed to make their distribution more regular before use in Eq. 2.

### SMC for POM calibration to distributions

Constructing a population of θ values that exhibits the estimated distribution *p*(**y**) is not trivial, given the complex relationship between the two encoded by Eq. 1. We defined *g*(θ) to be a probability density over the space θ and the population distribution of the model output M(θ) when θ is drawn according to this distribution we denote *h*(**y**|*g*(θ)), with the vertical bar | denoting conditioning. That is, *h*(**y**|*g*(θ)) is the density of M(θ) when θ ~ *g*(θ)

$$h(y|g(\theta ))=\underset{\Delta_{y}\to 0}{\text{lim}}\frac{1}{\prod_{k=1}^{N_{b}}\Delta_{b_{k}}}\int_{M(\theta )\in (y,y+\Delta_{y})}g(\theta )d\theta$$ (5)

where $\Delta_{y}=(\Delta_{b_{1}},\ldots ,\Delta_{b_{N_{b}}})$. Our problem is thus recast as finding the distribution *g*(θ) that produces *h*(**y**|*g*(θ)) that is as close as possible to *p*(**y**). The models sampled according to this optimal *g*(θ) will then exhibit outputs that reproduce the estimated distribution of outputs in the data.

If the “closeness” of *h*(**y**|*g*(θ)) and *p*(**y**) is measured in terms of the Kullback-Leibler divergence between the two distributions, the problem is an optimization problem

$$g*(\theta )=\;\text{arg}\;\underset{g(\theta )}{\text{min}}\int_{y}\text{ln}(\frac{h(y|g(\theta ))}{p(y)})h(y|g(\theta ))dy$$ (6)

The standard method for solving this type of problem is to follow a variational Bayes (VB) type of approach. VB is commonly used to produce parametric approximations of posterior distributions in Bayesian statistics [(*59*), chap. 11]. Applying VB to our problem would involve specifying some parametric distribution for *g*(θ)≡*g*(θ|φ), where φ are the parameters of the distribution. For example, if a multivariate normal distribution was adopted for *g*, φ would consist of a mean vector and covariance matrix. Using VB, Eq. 6 then reduces to finding the optimal parameters $\varphi *=\;\text{arg}\;{\text{min}}_{\varphi }f(\varphi )$, with the integral in Eq. 6 approximated by Monte Carlo integration taking *K* independent draws from *g*(θ|φ)

$$\int_{y}\text{ln}(\frac{h(y|g(\theta |\varphi ))}{p(y)})h(y|g(\theta |\varphi ))dy\approx \frac{1}{K}\sum_{k=1}^{K}\text{ln}(\frac{\hat{h}(y_{k}|g(\theta |\varphi ))}{p(y_{k})})=f(\varphi )$$ (7)

where $y_{k}=M(\theta_{k})$ and θ_*k*_ ~ *g*(θ|φ) for *k* = 1, …, *K*. For a particular φ, an estimate of *h*(**y**|*g*(θ|φ)), which we denote as $\hat{h}(y_{k}|g(\theta |\varphi ))$, could be obtained using a kernel density estimate as in Eq. 2 based on the set of simulated biomarker values ${\left\{y_{k}\right\}}_{k=1}^{K}$. There are several reasons why we did not adopt this approach: First, it requires us to specify a parametric form for *g*(θ); second, evaluating *f*(φ) is very expensive because it involves solving the model *K* times; and last, φ will be high-dimensional, leading to a difficult optimization problem.

Instead, we used an approach that is more pragmatic and effective in this application. First, we determined a collection of θ values (or models) that produced biomarker values ***y*** that have relatively high density with respect to the data density *p*(***y***). Then, we removed models from this collection in an iterative fashion so that the distribution of corresponding biomarker values that remained was even closer to *p*(**y**) (see the next section).

For the first step, we used SMC (*60*), following the use of the technique to construct POMs calibrated to ranges in data (*22*). This technique begins with a set of *N* particles and traverses them through a sequence of probability distributions by iteratively applying importance sampling, resampling, and move steps. We achieved this behavior by sampling from the sequence of distributions $h(\theta )\propto p{\left(M(\theta )\right)}^{\gamma }$, with γ ∈ [0,1]. It can immediately be seen that γ = 0 corresponds to the uniform distribution, which is very easy to sample if we specify some lower and upper limits for each component of θ, and that γ = 1 corresponds to a distribution proportional to p(M(θ)), which is potentially difficult to sample. Successively incrementing γ after each resample and move step until γ reaches 1 allows the complexity of the sampling problem to be introduced gradually. An important aspect of SMC is that it does not require the distributions in the sequence to be properly normalized. The full SMC algorithm is laid out in the Supplementary Materials.

The algorithm requires the use of traditional Markov chain Monte Carlo (MCMC) (*61*) steps to find unique locations for particles after each resampling step, and the particles must still represent samples from the current target distribution. This is achieved using the Metropolis-Hastings algorithm, which, in our setup, accepted or rejected any proposed particle moves according to

$$\text{Pr}(\text{accept})=\;\text{min}(1,\frac{{\left[p(M(\theta_{\text{new}}))\right]}^{\gamma }J(\theta_{\text{old}}|\theta_{\text{new}})}{{\left[p(M(\theta_{\text{old}}))\right]}^{\gamma }J(\theta_{\text{new}}|\theta_{\text{old}})})$$ (8)

where J is the jumping distribution that generates proposed moves of particles and, in our case, does not depend on the previous particle location [that is, $J(\theta_{\text{new}}|\theta_{\text{old}})=J(\theta_{\text{new}})$]. The jumping distribution used is a Gaussian mixture model built for a regularized version of the current of locations of all particles after a resampling step (see the “SMC algorithm” section in the Supplementary Materials). The number of MCMC steps performed after each resampling step was chosen adaptively (*62*).

The output of the SMC algorithm is a collection of samples ${\left\{\theta_{i}\right\}}_{i=1}^{N}$ from a distribution proportional to p(M(θ)). We note that the corresponding collection of output values ${\left\{y_{i}\right\}}_{i=1}^{N}$, where $y_{i}=M(\theta_{i})$, is not a sample from the distribution of interest, *p*(**y**). The reason for this is that we have not accounted for the nonlinear transformation, y=M(θ), to correctly convert the target distribution over the output space to the corresponding target distribution over the parameter space. However, given the fact that the transformation **y=M(θ)** is not analytic and not a one-to-one function, we suggest that it is not tractable to properly account for it, and it may not even be possible to find a distribution over θ that leads to a distribution of biomarker values consistent with *p*(**y**).

Nonetheless, we found that this approach led to a collection of parameter values that generated biomarker values with relatively high density under *p*(**y**). We used this collection as the starting point for our subsequent refinement process.

As an alternative to SMC, an MCMC approach could also be used directly to produce samples from a distribution proportional to p(M(θ)) using the same density (Eq. 2) and acceptance algorithm (Eq. 8) with γ = 1. We used a modern state-of-the-art MCMC sampler, DiffeRential Evolution Adaptive Metropolis (DREAM) (*63*), to verify our SMC algorithm and found that the two produced comparable results in terms of the distribution of the values of θ produced. The primary benefit of the SMC algorithm, when it came to the construction of our initial POM, was that the output of the algorithm is a set of unique samples from the distribution almost the same size as the number of particles, *N*, which was specified by the user. In contrast, MCMC approaches must be run an indefinite amount of time until the chain has been judged to have converged, producing a long chain of samples of initially unknown length. Moreover, these chains contain many repeated samples, and filtering out these repeats will also destroy the desired distribution.

### Further POM refinement

The approximate nature of the SMC calibration process encouraged further refinement of the constructed POMs to fully capture the statistical distributions seen in the data. This was achieved by selecting a subset of the population such that the new smaller set of models better exhibited the biomarker distributions seen in the data. To do this, first, a quantitative measure of how well a POM captured the distributions observed in the data was constructed. We used the Jensen-Shannon distance (JSD), a symmetric and finite version of the Kullback-Leibler divergence that remains a measure of the “distance” between two probability distributions. By labeling the two distributions *p*(**y**) and *q*(**y**), the JSD is given by

$$\text{JSD}\;={\left[\frac{1}{2}\int_{y}p(y)\text{ln}\;(\frac{p(y)}{\frac{1}{2}p(y)+\frac{1}{2}q(y)})dy+\frac{1}{2}\int_{y}q(y)\text{ln}\;(\frac{y}{\frac{1}{2}p(y)+\frac{1}{2}q(y)})dy\right]}^{1/2}$$ (9)

with the square root used to make the divergence measure a metric (*64*).

When the data are high-dimensional (say *N* ≥ 5), the “full” JSD between the multivariate joint distributions of the observations in the data set and those generated by a given POM is difficult to calculate accurately. Therefore, we instead used the distances between the marginal distributions for each of the biomarkers, JSD_*i*_, along with the distances between the bivariate distributions between all possible pairs of observation variables, JSD_*ij*_, to create a matrix, the norm of which serves as an approximate measure of fit, namely

$$\begin{array}{ccc} \rho ={\left||P|\right|}_{2} & & \\ P_{ij}={\text{JSD}}_{i} & \text{for}\;i=j & \\ ={\text{JSD}}_{ij} & \text{for}\;i\neq j & \left(i,j=1,\ldots ,N_{B}\right) \end{array}$$

The measure ρ takes into account how well the individual distributions of each observed variable are represented by a POM, along with some measure of how well it captures the dependency between these variables. We note that this is not necessarily the best measure of fit but uses more easily calculated divergences to produce a single value, allowing the use of the technique described below. JSD values used in the calculation of ρ, as integrals, were approximated using standard Riemann integration.

Our refinement process seeks to minimize ρ using a supplied POM. Here, we used POMs selected using SMC to improve fit with the data, although we note that POMs constructed using typical Monte Carlo sampling techniques (such as LHS and calibrated to the ranges of the data) could also be used as starting points for our refinement procedure. Minimization was achieved by trialing removal of individual models from the population (or reintroduction of removed models) and then accepting or rejecting them according to the Metropolis probability

$$\text{Pr}(\text{accept})=e^{-\Delta \rho /T}$$ (11)

where Δρ is the change in the overall divergence measure (Eq. 10) associated with the trialed removal/reintroduction and *T* is a parameter of the process that controls the likelihood of accepting unfavorable trial updates. This approach is very similar to the approach of simulated annealing (*65*), although we use a fixed value of *T* = 0.2 instead of gradually decreasing it. Every 1000 trial steps, we judged whether the choice of subpopulation was wandering too far away from the optimum by checking if ρ was more than 1% larger than the current best ρ value found and, if so, restarted the process back to the configuration corresponding to the best ρ value.

The only additional condition we used was that the size of the subpopulation of models could not fall below the number of data points, ensuring that the resulting population did not become small enough to lose meaning. If a larger population of models is desired, ρ (representing the energy of the system that is minimized over the course of the annealing process) can be replaced by a new expression in Eq. 11 that penalizes both higher values of ρ and small numbers of models in the population.

We saw (see Results) that the data for one biomarker, namely, the maximum upstroke velocity, took values not predicted by the CRN model in the search space, and strong correlations exhibited by the model were not seen in the data. This made it appealing to de-emphasize the contributions of this biomarker to the POM refinement process. This was achieved by creating a second divergence measure, $\hat{\rho }$, that is the two-norm of a modified version of the performance matrix **P**, with the row and column corresponding to the maximum upstroke velocity overwritten with zeroes, except for the diagonal element. Minimizing $\hat{\rho }$ instead allowed the distributions of the other biomarkers to be better fit by the refinement process, at the cost of producing POMs that did not strongly reflect the distribution of maximum upstroke velocities in the data. Given that we expected the maximum upstroke velocity to be the biomarker most subject to experimental measurement error in the data, we considered this a reasonable decision.

## Supplementary Material

http://advances.sciencemag.org/cgi/content/full/4/1/e1701676/DC1

## SUPPLEMENTARY MATERIALS

Supplementary material for this article is available at http://advances.sciencemag.org/cgi/content/full/4/1/e1701676/DC1 table S1. Summary statistics for the SR and cAF data sets are well recovered by the calibrated POMs. table S2. SMC with subsequent refinement produces POMs with very low divergence from the distributions in the data. fig. S1. Calibration to biomarker distributions, as opposed to their ranges, significantly reduces model bias for the cAF data set. fig. S2. Variability in the cAF data set is captured by a population of CRN models with varying current densities. fig. S3. Further variance in *I*_Na_ improves the realization of *dV*/*dt*_max_ values in the SR data set. fig. S4. Calibration to ranges fails to capture the morphological differences between SR and cAF atrial APs. fig. S5. Calibrating to data ranges does not identify all changes in ionic behavior associated with the cAF pathology. fig. S6. The distributions of parameter values selected for the SR and cAF POMs are distinct but regular. fig. S7. Variation of ±30% in current densities underestimates biomarker variance in the cAF data set.
