# Supplementary material for: Unlocking data sets by calibrating populations of models to data density: A study in atrial electrophysiology
Source: Sci Adv. 2018 Jan 10;4(1):e1701676. doi: 10.1126/sciadv.1701676 (PMC5770172; doi:10.1126/sciadv.1701676)
Supplement: http://advances.sciencemag.org/cgi/content/full/4/1/e1701676/DC1 [file supp_4_1_e1701676__index.html]

Science Advances | Science Advances

## Supplementary Materials

**This PDF file includes:**

- table S1. Summary statistics for the SR and cAF data sets are well recovered by the calibrated POMs.
- table S2. SMC with subsequent refinement produces POMs with very low divergence from the distributions in the data.
- fig. S1. Calibration to biomarker distributions, as opposed to their ranges, significantly reduces model bias for the cAF data set.
- fig. S2. Variability in the cAF data set is captured by a population of CRN models with varying current densities.
- fig. S3. Further variance in *I*Na improves the realization of *dV/dt*max values in the SR data set.
- fig. S4. Calibration to ranges fails to capture the morphological differences between SR and cAF atrial APs.
- fig. S5. Calibrating to data ranges does not identify all changes in ionic behavior associated with the cAF pathology.
- fig. S6. The distributions of parameter values selected for the SR and cAF POMs are distinct but regular.
- fig. S7. Variation of ±30% in current densities underestimates biomarker variance in the cAF data set.

Download PDF

**Files in this Data Supplement:**

- Adobe PDF - 1701676\_SM.pdf
