# Supplementary material for: Unlocking data sets by calibrating populations of models to data density: A study in atrial electrophysiology
Source: Sci Adv. 2018 Jan 10;4(1):e1701676. doi: 10.1126/sciadv.1701676 (PMC5770172; doi:10.1126/sciadv.1701676)
Supplement: http://advances.sciencemag.org/cgi/content/full/4/1/e1701676/DC1 [file 1701676_SM.pdf]

Science Advances

AAAS

advances.sciencemag.org/cgi/content/full/4/1/e1701676/DC1

Supplementary Materials for

Unlocking data sets by calibrating populations of models to data density: A study in atrial electrophysiology

Brodie A. J. Lawson, Christopher C. Drovandi, Nicole Cusimano, Pamela Burrage, Blanca Rodriguez, Kevin Burrage

Published 10 January 2018, *Sci. Adv.* **4**, e1701676 (2018)
DOI: 10.1126/sciadv.1701676

This PDF file includes:

- table S1. Summary statistics for the SR and cAF data sets are well recovered by the calibrated POMs.
- table S2. SMC with subsequent refinement produces POMs with very low divergence from the distributions in the data.
- fig. S1. Calibration to biomarker distributions, as opposed to their ranges, significantly reduces model bias for the cAF data set.
- fig. S2. Variability in the cAF data set is captured by a population of CRN models with varying current densities.
- fig. S3. Further variance in I_{Na} improves the realization of dV/dt_{max} values in the SR data set.
- fig. S4. Calibration to ranges fails to capture the morphological differences between SR and cAF atrial APs.
- fig. S5. Calibrating to data ranges does not identify all changes in ionic behavior associated with the cAF pathology.
- fig. S6. The distributions of parameter values selected for the SR and cAF POMs are distinct but regular.
- fig. S7. Variation of ±30% in current densities underestimates biomarker variance in the cAF data set.

Supplementary Materials

SR POMs (data)				
**Biomarker**	**Range**	**Mean**	**Std. Dev.**	**JSD**
APD_{90} (ms)	191−470 (193−467)	319 (318)	43 (44)	0.026
APD_{50} (ms)	7−215 (6−206)	142 (139)	40 (44)	0.066
APD_{20} (ms)	2−61 (2−63)	7 (7)	7 (8)	0.143
APA (mV)	77−120 (78−116)	95 (95)	7 (7)	0.021
RMP (mV)	-87−-65 (-87−-61)	-74 (-74)	4 (4)	0.059
V_{20} (mV)	-37−6 (-39−11)	-16 (-16)	6 (6)	0.041
dV/dt_{max} (V/s)	68−292 (48 −431)	156 (220)	34 (68)	0.426

cAF POMs (data)				
**Biomarker**	**Range**	**Mean**	**Std. Dev.**	**JSD**
APD_{90} (ms)	148−351 (141−349)	216 (216)	33 (35)	0.032
APD_{50} (ms)	33−168 (36−182)	101 (102)	28 (28)	0.040
APD_{20} (ms)	2−114 (4−82)	29 (30)	20 (18)	0.075
APA (mV)	81−119 (74−133)	102 (102)	7 (8)	0.075
RMP (mV)	-87−-68 (-90−-66)	-77 (-77)	3 (4)	0.073
V_{20} (mV)	-29−21 (-33−21)	-4 (-4)	10 (11)	0.049
dV/dt_{max} (V/s)	101−301 (40 −414)	189 (232)	34 (70)	0.361

table S1. **Summary statistics for the SR and cAF datasets are well recovered by the calibrated POMs.** Summary statistics for the POMs calibrated by minimising $\hat{\rho}$, for the biomarkers exhibited by SR and cAF atrial cells, as compared to the summary statistics for the experimental data itself (given in parentheses). Deviation in the marginal distributions of each biomarker are specified in terms of the Jensen-Shannon distance, calculated using equation (9, main document). The statistical variation of the data is seen to be well captured in both cases, apart from the maximum upstroke velocity, which also manifests in a large JSD value.

**Population of Models**	**SR data**		**cAF data**	
	ρ	$\hat{\rho}$	ρ	$\hat{\rho}$
LHS, matched to ranges	2.76	2.41	2.74	2.42
SMC, matched to distributions	1.95	1.41	2.16	1.74
SMC subpopulation, minimising ρ	1.36	0.68	1.21	0.70
SMC subpopulation, minimising $\hat{\rho}$	1.47	0.49	1.28	0.57
Additional I_{Na} variability, minimising ρ	0.80	0.55	0.75	0.58
$\pm 30\%$ variability, minimising $\hat{\rho}$	1.79	1.01	1.77	1.19

table S2. **SMC with subsequent refinement produces POMs with very low divergence from the distributions in the data.** Comparison of the ability of different POMs to capture the between-subject variability in two experimental datasets, as provided by the divergence measures ρ and $\hat{\rho}$. Lower ρ values indicate a better fit to the distributions, demonstrating a significant gain from both the SMC and from choosing an optimal subpopulation. The results for the additional hypotheses regarding the present variability are also listed, with additional variability in I_{Na} providing the best overall fit to all biomarkers, and $\pm 30\%$ variability in channel conductances seen to be insufficient to predict the full variability in the biomarker data. For reference, a complete divergence between data and POM would produce $\rho_{\text{max}} = 5.83$ and $\hat{\rho}_{\text{max}} = 5.00$.

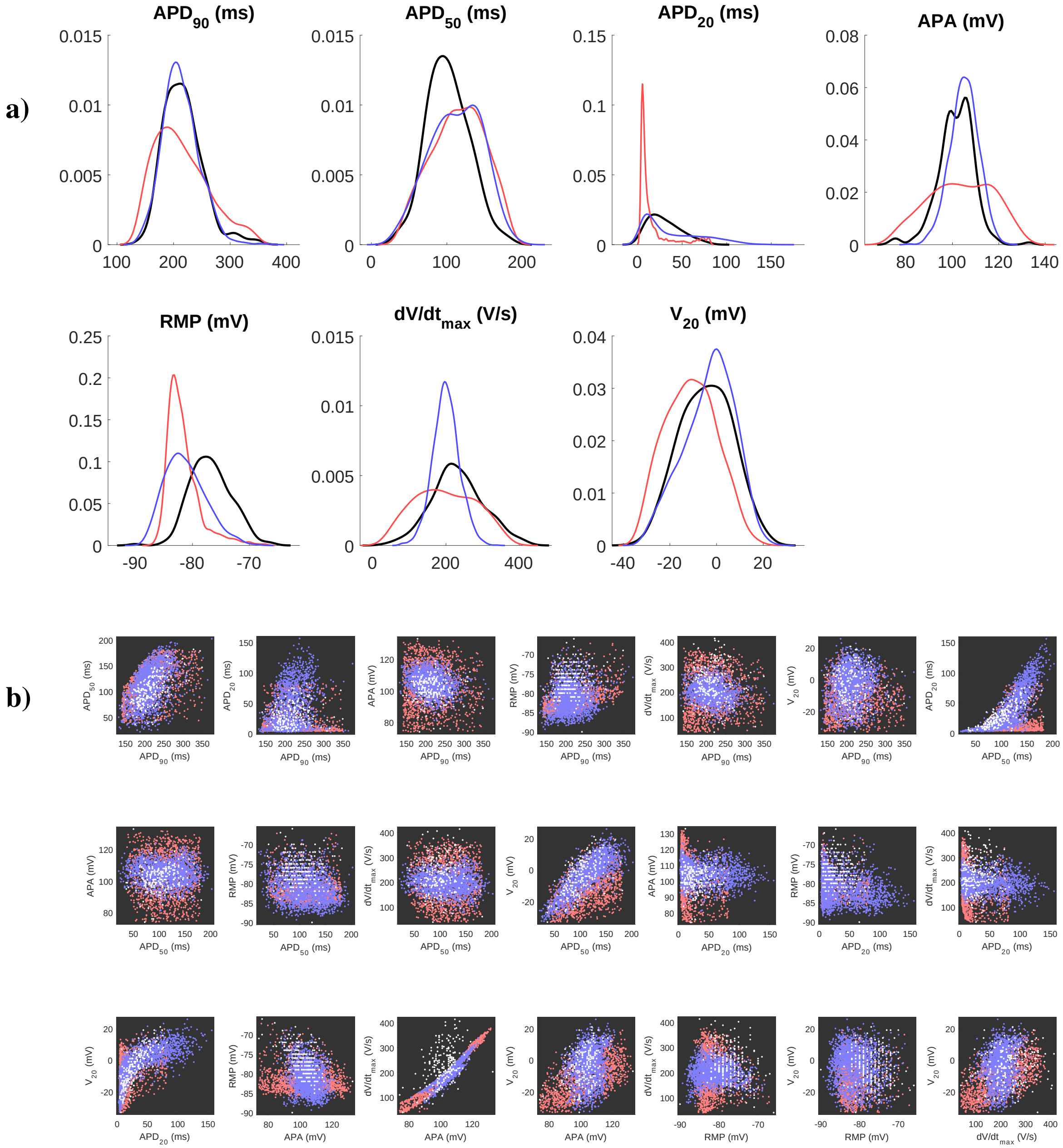

fig. S1**. Calibration to biomarker distributions, as opposed to their ranges, significantly reduces model bias for the cAF dataset. a)** Marginal distributions of the biomarkers in the cAF dataset (black) and POMs calibrated to biomarker distributions using the SMC algorithm (blue) or calibrated to biomarker ranges using LHS (red). SMC for distributional calibration is seen to provide a significant improvement in agreement with the data. **b)** Pairwise scatterplots of each unique pair of biomarkers in the SR dataset (white) and the POMs constructed using SMC matched to distributions (blue) and LHS matched to ranges (red). The SMC-generated POM demonstrates good localisation to the dense regions in the data, but clearly requires further calibration. An obvious correlation between APA and dV/dt_{max} is exhibited by the model, regardless of the sampling method used, but this correlation is not present in the data.

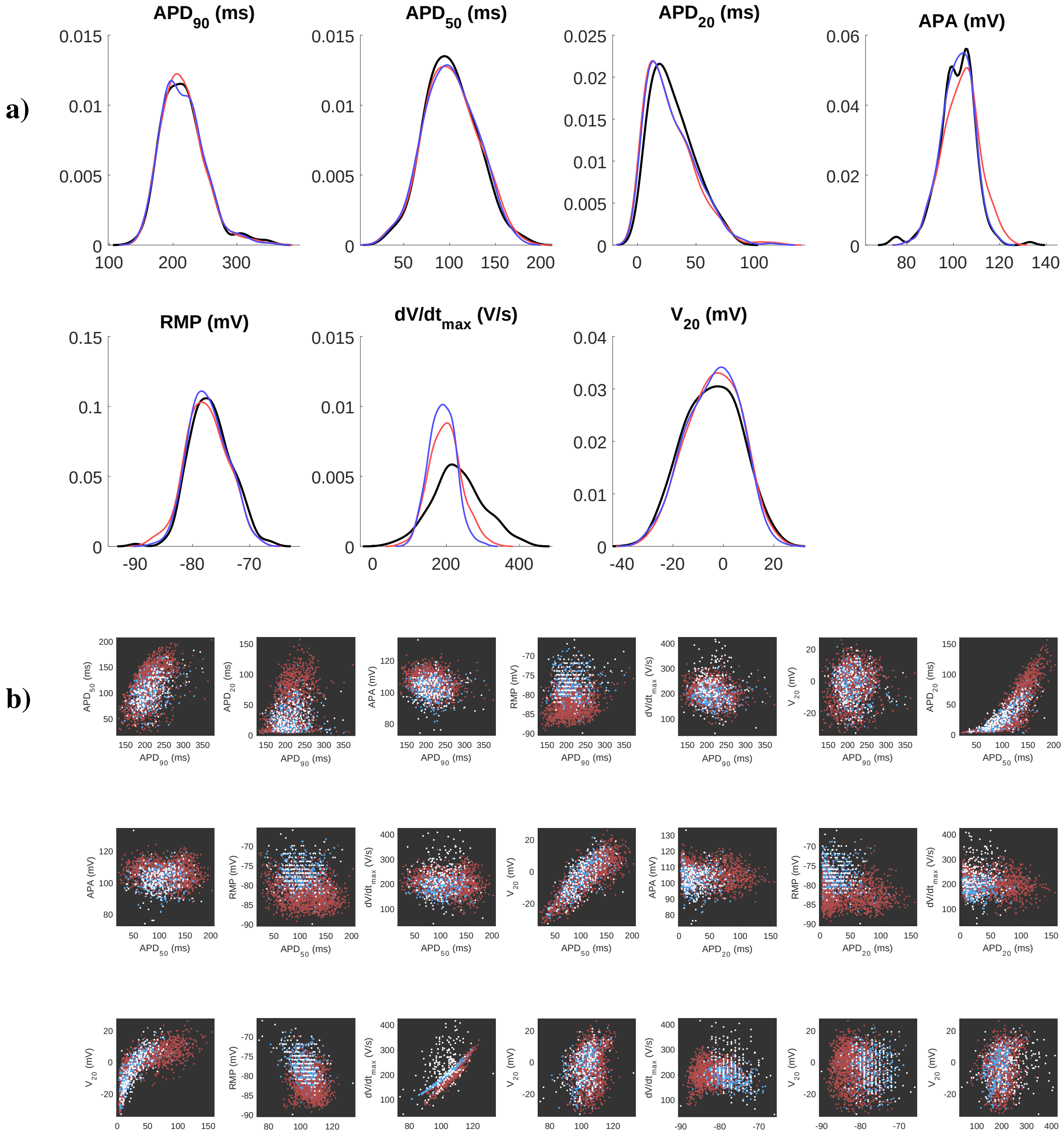

fig. S2. **Variability in the cAF dataset is captured by a population of CRN models with varying current densities.** **a)** Marginal distributions of the biomarkers in the cAF dataset (black) and the POMs constructed using SMC followed by simulated annealing to minimise ρ (red) or $\hat{\rho}$ (blue). Matching of the univariate biomarker distributions is slightly less well achieved than in the case of the SR datsaet, but the calibration process is clearly very successful and the trends in the data captured by the constructed POM. **b)** Pairwise scatterplots of each unique pair of biomarkers in the cAF dataset (white) and the models from the SMC-generated POM that are accepted (light blue) or rejected (dark red) in the process of minimising $\hat{\rho}$. Only the spread of, and correlations with, dV/dt_{max} are not captured very well by the final POM.

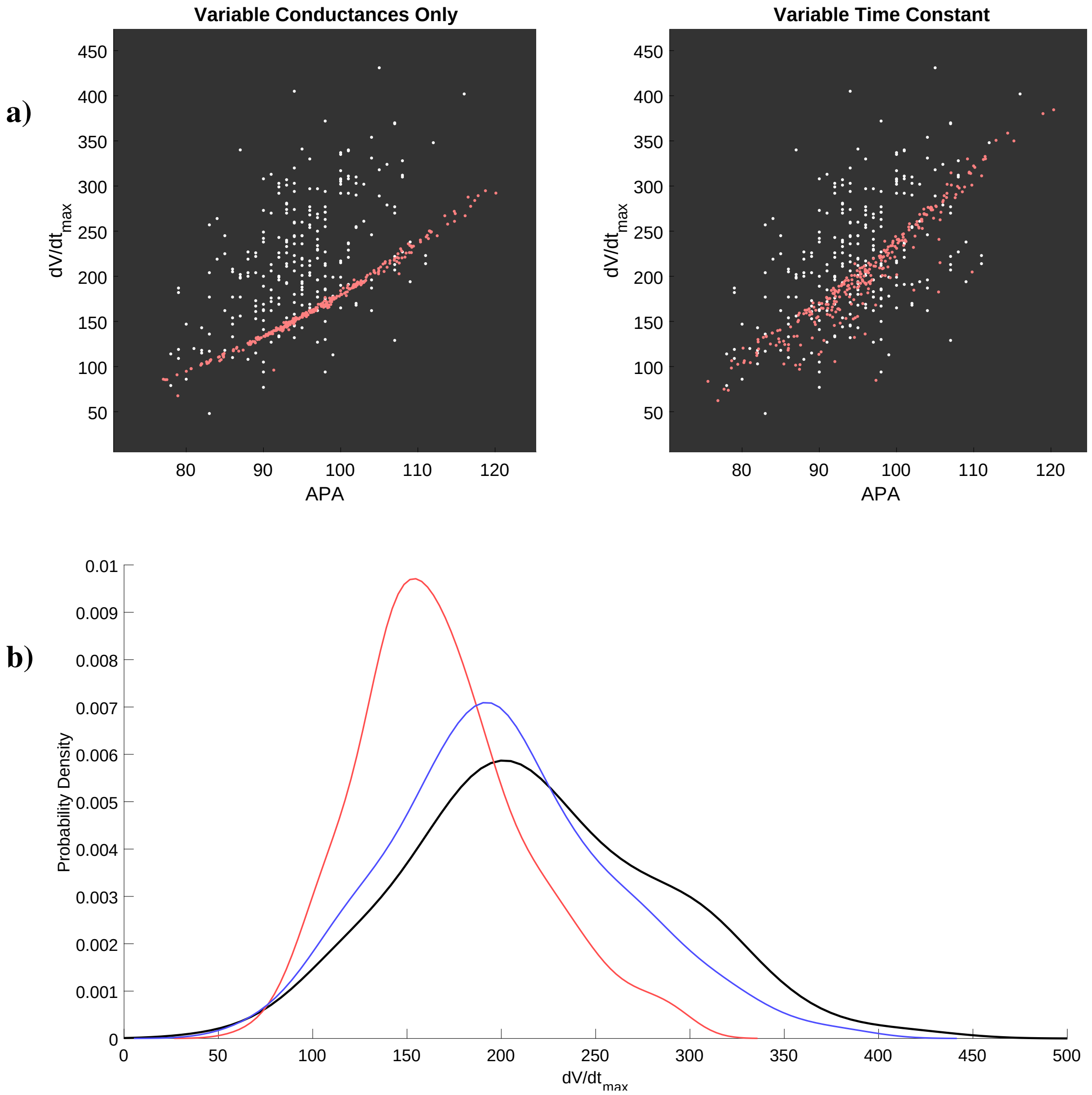

fig. S3. **Further variance in I_{Na} improves the realisation of dV/dt$_{\mathbf{max}}$ values in the SR dataset. a)** Pairwise scatterplot of APA and dV/dt$_{max}$ values in the SR dataset (white) and those accepted by distribution-calibrated POMs minimising ρ (red). Allowing variance in the time constant significantly reduces the correlation between these two biomarkers in the POM, better realising the spread of the data. **b)** Marginal distribution of dV/dt$_{max}$ values in the SR dataset (black) and the calibrated POMs varying only current conductances (red) or with additional variance in I_{Na} conductance and inactivation time (blue). This additional variance allows our calibrated POM to almost capture the marginal distribution of dV/dt$_{max}$ values were the original POM fails.

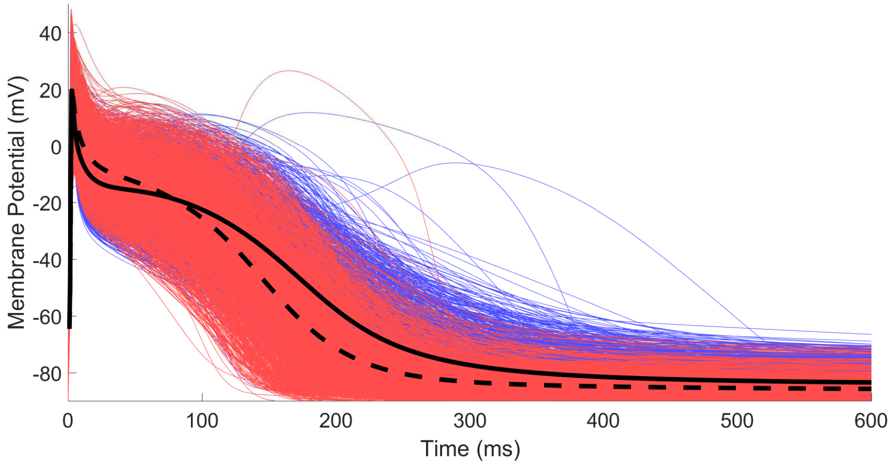

fig. S4**. Calibration to ranges fails to capture the morphological differences between SR and cAF atrial action potentials.** Atrial action potentials produced by simulation of the populations of CRN models calibrated to the ranges of biomarker data for patients exhibiting sinus rhythm (blue) and chronic atrial fibrillation (red). Also displayed are the average of all traces for the sinus rhythm (solid) and atrial fibrillation (dashed) populations. Differences in AP morphology are far less pronounced than those observed using calibration to distributions, and a small number of simulated APs appear unphysical.

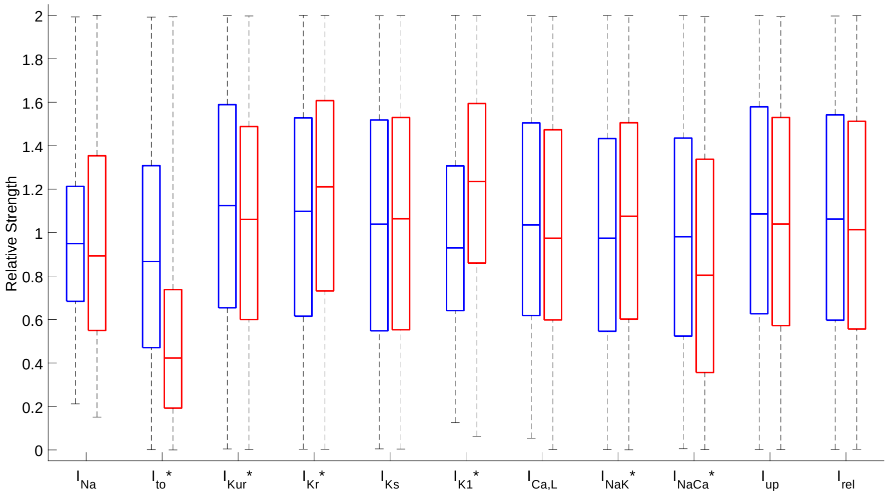

fig. S5. **Calibrating to data ranges does not identify all changes in ionic behaviour associated with the cAF pathology.** Boxplot of $\boldsymbol{\theta}$ values composing the POMs calibrated to the ranges of the SR (blue) and cAF (red) datasets using LHS. Values are expressed in relation to the base parameter values for the CRN model. Current densities that show statistically significant differences ($p < 0.001$ from the Mann-Whitney U test) are indicated with a *. Although some currents are identified as statistically different between SR and cAF, the effect sizes are small in almost all cases. The POMs do show the expected decrease in I_{to} but predict only a minor decrease in I_{Kur} and do not identify the decrease in I_{CaL}. I_{NaCa} is suggested to decrease in cAF, in contrast to experimental evidence.

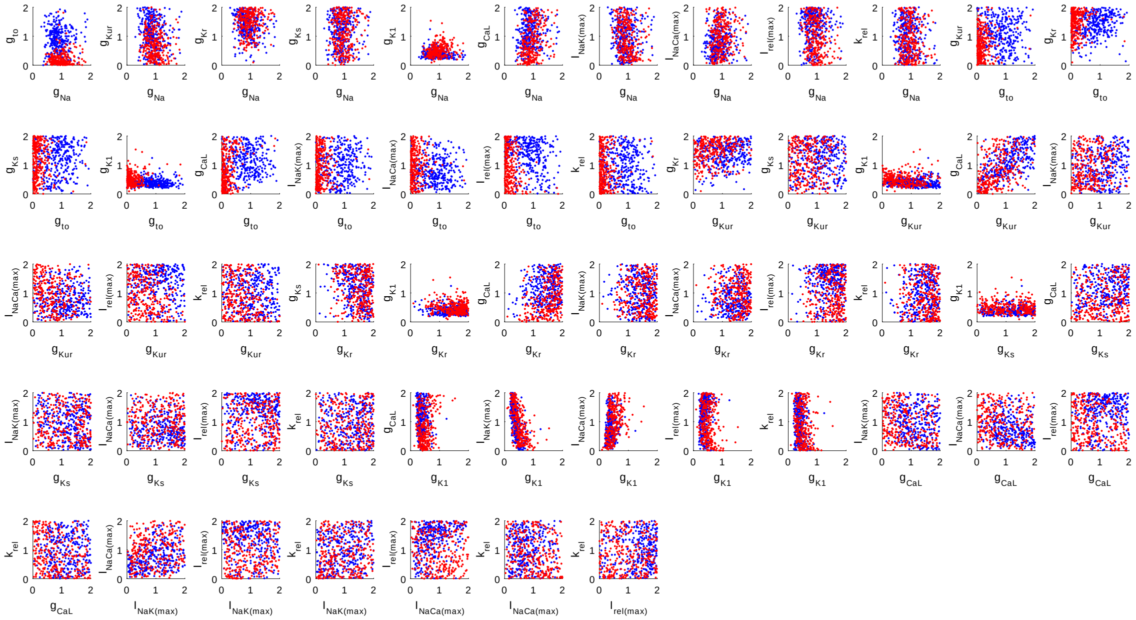

fig. S6**. The distributions of parameter values selected for the SR and cAF POMs are distinct, but regular.** Pairwise scatterplots of the parameter values selected for POMs calibrated to the SR (blue) and cAF (red) datasets, expressed in terms of the proportion of the base values for parameters in the CRN model. Clear differences in the two distributions can be observed, but neither POM exhibits obvious patterns of correlation in any pair of parameters, nor is there evidence of bimodality. These properties are important when reducing a POM back to a single representative model.

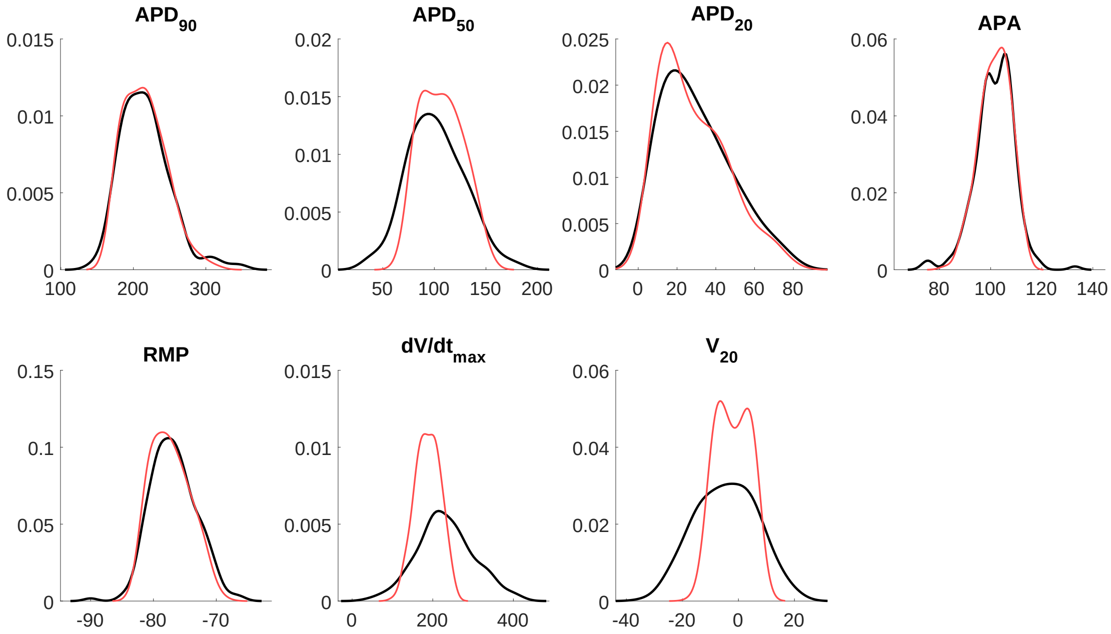

fig. S7. **Variation of $\pm 30\%$ in current densities underestimates biomarker variance in the cAF dataset.** Marginal distributions of the biomarkers in the cAF dataset (black) and distribution-calibrated POM using $\pm 30\%$ variance in ion channel conductances (red). A reduced search space is still able to recover the general distributions of all biomarkers except for dV/dt_{max} and V_{20}, with the extent of variation in APD_{50} also significantly underestimated.

Algorithm 1 SMC algorithm for construction of a POM fitted to an underlying distribution of biomarkers, $p(\mathbf{y})$.

▷ Initialise particles
Set $i = 0$
while $i \leq N_{\text{parts}}$ **do**
    Select a random $\boldsymbol{\theta}$ from the search space and calculate $\mathbf{y} = \mathcal{M}(\boldsymbol{\theta})$
    if action potential not rejected (see Materials and Methods) **then**
        Set $i = i + 1$
        Store particle location in parameter space, $\boldsymbol{\theta}_i$ and biomarkers, $\mathbf{y}_i$
        Store particle likelihood, $\mathcal{L}_i = p(\mathbf{y_i})$
    end if
end while

▷ Gradually increment γ until the true distribution is sampled
Set $\gamma = 0$
while $\gamma < 1$ **do**

    ▷ Check if current particles sufficiently reproduce the desired distribution
    if ESS$(\gamma, 1) \geq N_{\text{parts}}/2$ **then**
        Set $\gamma = 1$
    else
        Find γ' such that ESS$(\gamma, \gamma') = N_{\text{parts}}/2$
    end if

    ▷ Resample particles according to the new distribution
    Calculate normalised weights for particles, $w_i = \mathcal{L}_i{}^{(\gamma'-\gamma)} / \sum_{j=1}^{N_{\text{parts}}} \mathcal{L}_j{}^{(\gamma'-\gamma)}$
    Resample particle locations $\boldsymbol{\theta} \sim$ Multinomial(w)
    Update $\gamma \to \gamma'$

    ▷ Attempt to remove particle duplications via MCMC move steps
    Construct the jumping distribution, $\mathcal{J}(\boldsymbol{\theta}) =$ BUILDJUMPDIST$(\boldsymbol{\theta})$
    Update particle locations, $[\boldsymbol{\theta}, \mathbf{y}, \text{acc}]$ = MCMCMOVE$(\boldsymbol{\theta}, \mathbf{y})$
    Determine optimal number of MCMC iterations, $R = \text{ceil}\left(\frac{\ln 0.05}{\ln(1-\text{acc})}\right)$
    for $i = 1$ to min$(R - 1, 29)$ **do**
        $[\boldsymbol{\theta}, \mathbf{y}, \sim] =$ MCMCMOVE$(\boldsymbol{\theta}, \mathbf{y}, \mathcal{J}(\boldsymbol{\theta}))$
    end for

end while

Algorithm 2 Ancillary functions used by the SMC algorithm

function ESS(γ, γ')
  Calculate particle weights, $w_i = \mathcal{L}_i{}^{(\gamma'-\gamma)} / \sum_{j=1}^{N_{\text{parts}}} \mathcal{L}^{(\gamma'-\gamma)}$
  Return estimated sample size, $\text{ESS} = 1/\sum_{j=1}^{N_{\text{parts}}} w_j{}^2$
end function

function $\mathcal{J}(\boldsymbol{\theta}) = \text{BuildJumpDist}(\boldsymbol{\theta})$
  ▷ Regularise the marginal distributions of $\boldsymbol{\theta}$
  Scale particle locations to $[0, 1]$, $\boldsymbol{\phi}_i = \frac{\boldsymbol{\theta}_i - \boldsymbol{\theta}_{\text{min}}}{\boldsymbol{\theta}_{\text{max}} - \boldsymbol{\theta}_{\text{min}}}$
  Fit a beta distribution to the values of $\boldsymbol{\phi}$.
  Use this to find an optimal mixture of two beta distributions, $f(\boldsymbol{\phi})$
  Use the cdf of the beta mixture, $\mathbf{u}_i = F(\boldsymbol{\phi}_i)$ to obtain approximately uniformly distributed particles
  Transform these into normally distributed particles, $\mathbf{z} = \text{norminv}(\mathbf{u})$
  ▷ Jumping dist. is Gaussian mixture model on regularised distributions
  Fit a mixture of three Gaussians to particle $\mathbf{z}$'s using MATLAB's *fitgmdist*
  Store the Gaussian mixture model, $\mathcal{J}(\mathbf{z})$
  Calculate and store $\mathcal{J}(\mathbf{z})$ for all particles
end function

function $[\boldsymbol{\theta}, \mathbf{y}] = \text{MCMCMove}(\boldsymbol{\theta}, \mathbf{y}, \mathcal{J}(\boldsymbol{\theta}))$
  for $i = 1$ to N_{parts} **do**
    Propose $\mathbf{z}'_i \sim \mathcal{J}(\mathbf{z})$
    Transform $\mathbf{z}'_i$ back to $\boldsymbol{\theta}'_i$
    Evaluate the model, $\mathbf{y}'_i = \mathcal{M}(\boldsymbol{\theta}'_i)$
    ▷ Accept or reject according to Metropolis-Hastings algorithm
    Generate a uniform random number $r \sim [0, 1]$
    if $r < \min\left(1, \frac{[p(\mathbf{y}'_i)]^\gamma \mathcal{J}(\boldsymbol{\theta}_i)}{[p(\mathbf{y}_i)]^\gamma \mathcal{J}(\boldsymbol{\theta}'_i)}\right)$ **then**
      Update $\boldsymbol{\theta}_i \to \boldsymbol{\theta}'_i$, $\mathbf{y}_i \to \mathbf{y}'_i$
    end if
  end for
end function
